# Microstructure and stress mapping in 3D at industrially relevant degrees of plastic deformation

**DOI:** 10.1038/s41598-024-71006-0

**Published:** 2024-08-30

**Authors:** Axel Henningsson, Mustafacan Kutsal, Jonathan P. Wright, Wolfgang Ludwig, Henning Osholm Sørensen, Stephen A. Hall, Grethe Winther, Henning Friis Poulsen

**Affiliations:** 1 Division of Solid Mechanics, Lund University, Ole Römers Väg 1, Lund, Sweden; 2 Department of Physics, Technical University of Denmark, Kongens Lyngby, Denmark; 3 European Synchrotron Radiation Facility, 71 Avenue des Martyrs, CS40220, 38043 Grenoble Cedex 9, France; 4 INSA Lyon, CNRS, MATEIS UMR5510, Universite Lyon I, 69621 Villeurbanne, France; 5 Xnovo Technology ApS, Galoche Allé 15, 1st Floor, 4600 Køge, Denmark; 6 Department of Civil and Mechanical Engineering, Technical University of Denmark, Kongens Lyngby, Denmark

**Keywords:** Metals and alloys, Microscopy

## Abstract

Strength, ductility, and failure properties of metals are tailored by plastic deformation routes. Predicting these properties requires modeling of the structural dynamics and stress evolution taking place on several length scales. Progress has been hampered by a lack of representative 3D experimental data at industrially relevant degrees of deformation. We present an X-ray imaging based 3D mapping of an aluminum polycrystal deformed to the ultimate tensile strength (32% elongation). The extensive dataset reveals significant intra-grain stress variations (36 MPa) up to at least half of the inter-grain variations (76 MPa), which are dominated by grain orientation effects. Local intra-grain stress concentrations are candidates for damage nucleation. Such data are important for models of structure-property relations and damage.

## Introduction

The utilization of polycrystalline metals and alloys is widespread across various industries such as modern electronics, automotive, construction, aerospace, and energy. Notably, through plastic deformation of a polycrystalline specimen, a permanent alteration of its shape with a spectrum of macroscopic properties can be effectively controlled. These properties encompass mechanical strength, hardness, and ductility, alongside electrical and thermal conductivity, as well as magnetic coercivity and permeability. To elucidate the mechanisms behind the evolution of these macroscopic properties with plasticity, an examination of the microscopic interplay both between and within the single crystal grains constituting the polycrystalline aggregate is imperative. This interplay is characterized by the local crystal orientation and stress state. Understanding this interplay across various length scales is pivotal for addressing fundamental scientific inquiries related to damage, creep, deformation twinning, and notably, the correlation between strength and processing. Consequently, there exists a significant demand for a quantitative mapping of the 3D microstructural and stress development of the polycrystal in response to external forces. Moreover, from an engineering safety standpoint, mapping plastic deformations at both inter- and intra-grain length scales is critical as they precede component failure.

Electron microscopy is widely employed to map the microstructure of metals. Transmission electron microscopy (TEM) enables nanometer-resolution 3D mapping of crystal orientation and defect structures within thin films^1–3^ and micro-pillars^4^. Concurrently, electron backscatter diffraction (EBSD) combined with serial sectioning facilitates the mapping of local orientation within extensive volumes^5,6^. However, these techniques fall short in tracking the dynamic evolution of the microstructure *in situ* in a manner representative of bulk behavior. Similarly, stress mapping^7,8^ faces challenges due to geometrical constraints arising from the limited penetration power of electrons. In contrast, X-ray diffraction based imaging methods such as 3D X-ray Diffraction^9^, Diffraction Contrast Tomography^10,11^ and lab-DCT^12^ excel in producing voxelated maps of the lattice orientation in deformed metals^13,14^ in mm-sized samples^15^ comprising up to tens of thousands of grains^16,17^. Nevertheless, the complementary mapping of the intra-grain stress^18^ has proven elusive, due to the increasing overlap of diffraction patterns from different parts of the sample as deformation progresses. Such maps are critically important in guiding and validating the modeling of plasticity and failure.

Introducing a monochromatic beam raster scanning modality, scanning 3DXRD (S3DXRD), reduces these limitations. By adapting reconstruction algorithms from tomography, complimentary mappings of intra-grain residual orientation and stress have been made^19–21^. This advancement has been further augmented through the use of conical slits^22^, which physically reduce the diffracting gauge volume^23,24^. As examples of results, residual stresses have been mapped in a plastically deformed titanium sample at $7$% tensile deformation^25^ and in a low carbon steel at $5.1$% tensile deformation^23^. However, to our knowledge, no 3D bulk reconstructions of intra-grain stress have been achieved at higher levels of deformation. In contrast, industrially relevant processes for metals, such as rolling, extrusion, drawing and forging typically require medium to high levels of deformation (10-90 %). Likewise, in simple tensile testing, most ductile metals may be elongated 20-40% before the onset of failure.

## Aim of the study

In order to push the deformation limit of the nondestructive 3D stress mapping, here, we present a novel reconstruction algorithm designed to address the challenges posed by diffraction spot overlap. Leveraging spatial correlation within the orientation field, this algorithm effectively mitigates noise. We demonstrate its use on a coarse-grained aluminum alloy subject to $>30$% tensile deformation in an S3DRXD set-up at the European Synchrotron Radiation Facility (ESRF). The sample is deformed to its ultimate tensile strength (UTS) where plastic deformation localizes and damage is initiated. We mapped the orientation and stress tensor in $\approx 250,000$ voxels in a volume of $440 \times 440 \times 9 \mu$m$^{3}$ in about 8 hours. The reconstruction revealed intra-grain misorientations exceeding $10^{\circ }$ (see fig. S7) along with stress localization on several length scales. Such comprehensive data offer a unique avenue for guiding, optimizing, and validating multi-length scale models. The data can be interfaced directly to voxelated simulation models (similar to, for example^26^), or statistical correlations between microstructural and stress variables can be extracted from the extensive dataset.

## Methods

**Figure 1:**
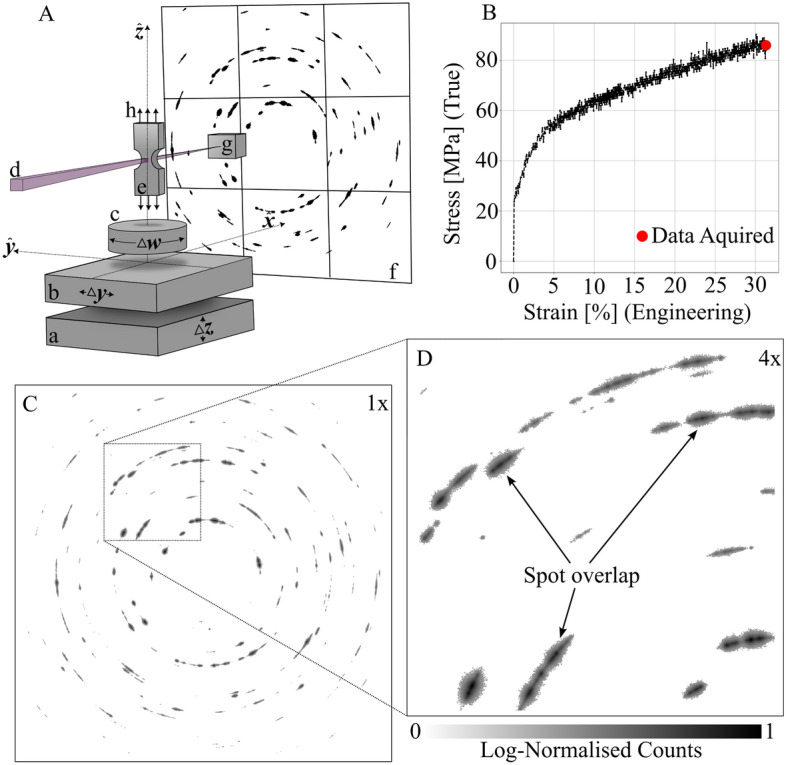
Experimental principle (**A**) Schematic of the X-ray diffraction imaging setup. The sample is scanned in directions $\varvec{\hat{y}}$ and $\varvec{\hat{z}}$ (a,b) and rotated (c) while probed with a focused X-ray beam (d). The diffraction signal from the notched sample (e) is acquired by an area detector (f,g) is a beamstop and (h) illustrates the tensile load pattern. (**B**) The stress-strain curve with indication of the ultimate tensile strength ($\sim 85$ MPa) where imaging was performed. (**C**) Diffraction pattern acquired while integrating over a $\Delta \omega =1^{\circ }$ sample rotation interval. (**D**) Zoom-in. Plastic deformations gave rise to peak arcing around the diffraction rings (**C**) and diffraction spot overlap (**D**).

The setup is illustrated schematically in Fig. 1A. The sample was notched to enable mapping at the precise location of the macroscopic stress localization. Following *in situ* tensile deformation, cf. Figs. 1B and S2, the sample remained under load and underwent tomographic raster scanning in steps of 3$\mu$m by moving a $~450 \times 450 \times 9 \mu$m$^3$ volume of interest across a 3$\mu$m $\times$ 3$\mu$m X-ray microbeam while rotating the diffractometer. The plastic deformation at this deformation level is evident through substantial spot overlap in the diffraction images and peak spreading around the diffraction rings, as depicted in Fig. 1C,D. For further details on the materials and X-ray methodology, see Supplementary Information.

Using algorithms from computed tomography, the illuminated sample volume was partitioned into 247,956 voxels (see fig. S3)^27^. For each voxel, crystal orientations and strains consistent with the diffraction data were determined using ImageD11^28^ and algorithms similar to those presented in^29–36^. This resulted in an orientation-strain map which listed the best candidate crystal lattices found for each voxel in the sample, with each voxel being treated independently of its neighbors. These local orientations and strains represent a multi-channel sampling of possible solutions to the inversion problem of retrieving the microstructure from the data. Due to the high dimension of the solution space, any approach reliant on trial-and-error optimization becomes computationally impossible.

For deformations much smaller than those presented in this study, where diffraction spot overlap remains limited, grain shapes can be reconstructed from diffracted intensities using tomographic methods^19,37–41^. Subsequently, once the grain shapes are determined, the strain-orientation field within individual grains can be inferred by solving another, modified, tomographic reconstruction problem^42^. However, for industrially relevant plastic deformation, diffraction peaks broaden and exhibit multiple local maxima, rendering tomographic grain shape reconstruction unfeasible.

In addition to tomographic reconstruction approaches, point-wise fitting methods have emerged, avoiding the use of diffracted intensities^23,34,35,43,44^. These methods reconstruct a strain-orientation map by selecting, for each voxel, the orientation with the highest completeness (i.e. the ratio of the number of observed diffraction peaks to expected peaks). While these methods theoretically produce a strain-orientation map at various deformation levels, the presence of erroneously scattered orientations increases with diffraction peak overlap. Consequently, distinguishing among solutions within the multi-channel orientation-strain map becomes challenging, particularly at elevated levels of plastic deformation.

**Figure 2:**
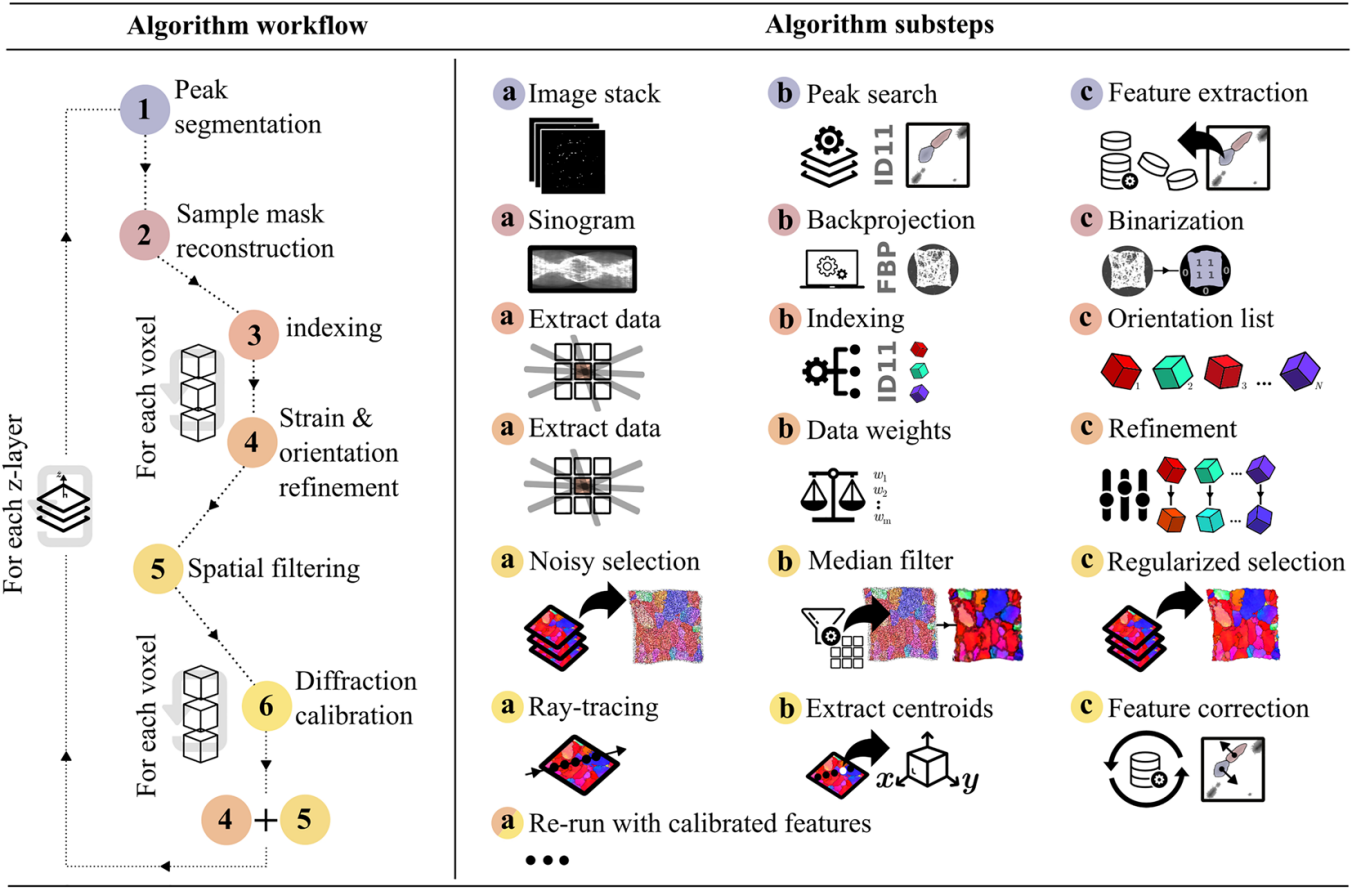
Algorithm overview. A voxelated strain-orientation map is reconstructed by following the algorithm workflow presented to the left. In each of the numbered workflow steps, a series of algorithm substeps are executed in alphabetic order, as illustrated to the right. In summary; for each scanned $z$-layer, diffraction peaks are segmented (1a–1c) and used to reconstruct a primary shape of the sample (2a–2c). The centroids of the segmented diffraction peaks are then used to generate (3a–3c) and refine (4a–4c) lists of candidate orientations and strains for each individual voxel in the sample. From the generated solution space, a primary, noisy, strain-orientation map is selected by considering the voxel completeness (5a). Next, a median orientation filter is applied to the primary selection (5b), and the original solution space is revisited to make a new, regularized selection (5c). The resulting strain-orientation map suppresses spatial noise and can be used with ray-tracing to compute approximate diffraction origins in sample space (6a,6b). After correcting the diffraction peak centroid data by the computed diffraction origins (6c) and propagating this correction into the strain-orientation fit (4a–4c) a final regularized selection of strain-orientation map is made (5a–5c). Iterating this procedure over all $z$-layers in the sample volume produces a 3D voxelated strain-orientation map. The six components presented in the algorithm workflow to the left are further detailed in the supplementary materials^27^.

To address these challenges, we introduce a new reconstruction algorithm. Our framework incorporates *a priori* knowledge of the spatial correlation of orientations by employing median filters directly on inverse pole figure space. Each voxel in the reconstructed volume generated an independent list of candidate lattice orientations by running the indexing code of ImageD11^28^ locally, inputting the sub-set of diffraction data that featured x-ray illumination of the voxel. By maximizing completeness and simultaneously enforcing spatial correlation between the orientations of neighboring voxels, we overcome the challenges posed by diffraction spot overlap. The overall workflow of our algorithm, along with its individual substeps, is illustrated in Fig. 2. As outlined in Fig. 2, steps 5a-5c, our algorithm enforces orientation correlation between neighboring voxels by first making a noisy orientation selection, based solely on voxel completeness, and then, as a secondary step, computing the median orientation in a neighborhood around each voxel. The original, multi-channel voxel volume is then inquired at each voxel location, searching for the orientation candidate that has the smallest misorientation compared to the median filtered orientation map. Consequently, we can efficiently search the multi-channel orientation-strain map for a solution, even at industrially relevant degrees of plastic deformation.

On the intra-grain scale, when the crystals deform, the existence of spatial correlation in the orientation field is fundamentally predicted by continuum mechanics. The median orientation filter is introduced to exploit this fact as a regularization, and its size will directly affect the achievable spatial resolution of the reconstruction. A reduction in filter size will therefore lead to less blurring in the orientation field, allowing for sharper spatial features to be reconstructed. On the other hand, reducing the size of the filter weakens the regularization and introduces more noise into the reconstructed field. In the supplementary materials^27^, a model is presented for the direct estimation of experimental error on the position of the grain boundaries using a 9$\mu$m $\times$ 9$\mu$m median filter (6 $\times$ 6 voxels). The standard deviation of this spatial error is found to be less than the beam size.

We implemented our algorithm separately for each $z$-layer of the volume and then stacked the solutions to construct a 3D grain volume. For an in-depth discussion of all algorithm components see supplementary materials^27^.

## Results

**Figure 3:**
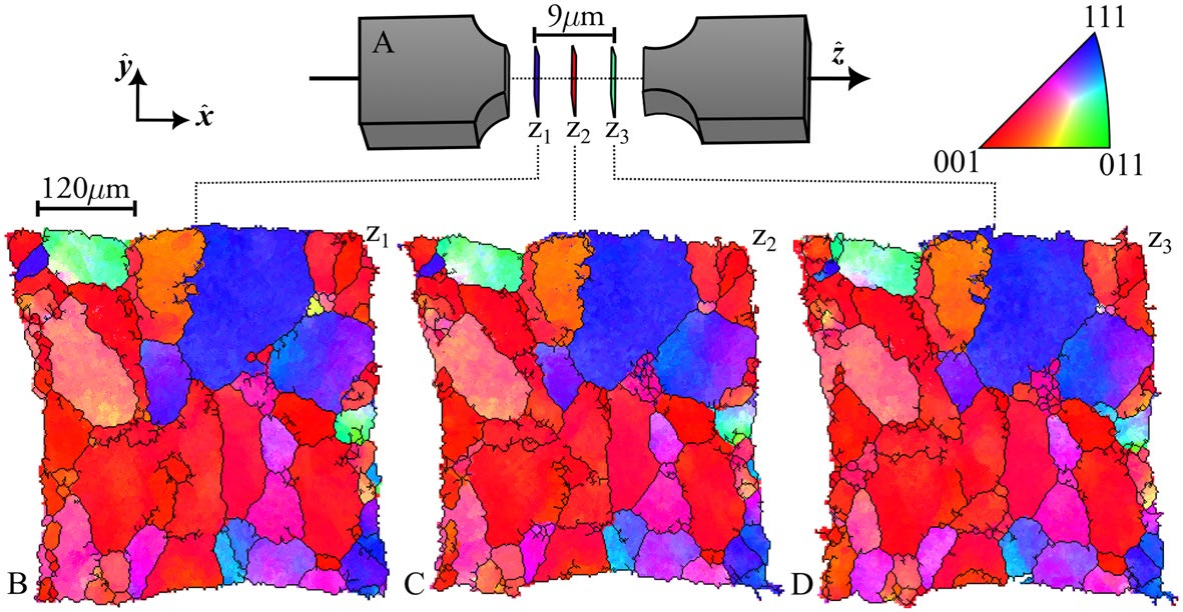
Reconstructed orientation maps. (**A**) Schematic of the aluminum sample highlighting the three slices (z1, z2 and z3) scanned, evenly spaced by 3 $\mu$m in $z$ and located at the sample notch center. (**B**–**D**) The orientation maps. The color map (top right) indicates the inverse pole figure (IPF) illustrating which crystallographic plane normal aligns with the tensile loading axis ($\varvec{\hat{z}}$). The maps have been overlaid with grain boundaries (black lines) defined by a local misorientation threshold of $4 ^{o}$.

The reconstructed orientation field is shown in Fig. 3. From this grain and sub-grain boundaries were identified by setting thresholds on the local mis-orientation (see figs. S4-S5)^27^. A plot of the average orientations of the resulting 60 grains (fig. S6) revealed the absence of grains with the tensile axis ($\varvec{\hat{z}}$) oriented along the crystallographic (011) direction - as expected after tensile deformation. Intra-grain misorientations reaching up to 10$^{\circ }$ were identified (fig. S7) which is to be compared to misorientations in the range of 1.5-3$^{\circ }$ reported in previous S3DXRD studies^23,25^. In the supplementary materials^27^, a model is presented for the direct estimation of experimental error on the position of the grain boundaries. The standard deviation is found to be less than the beam size.

**Figure 4:**
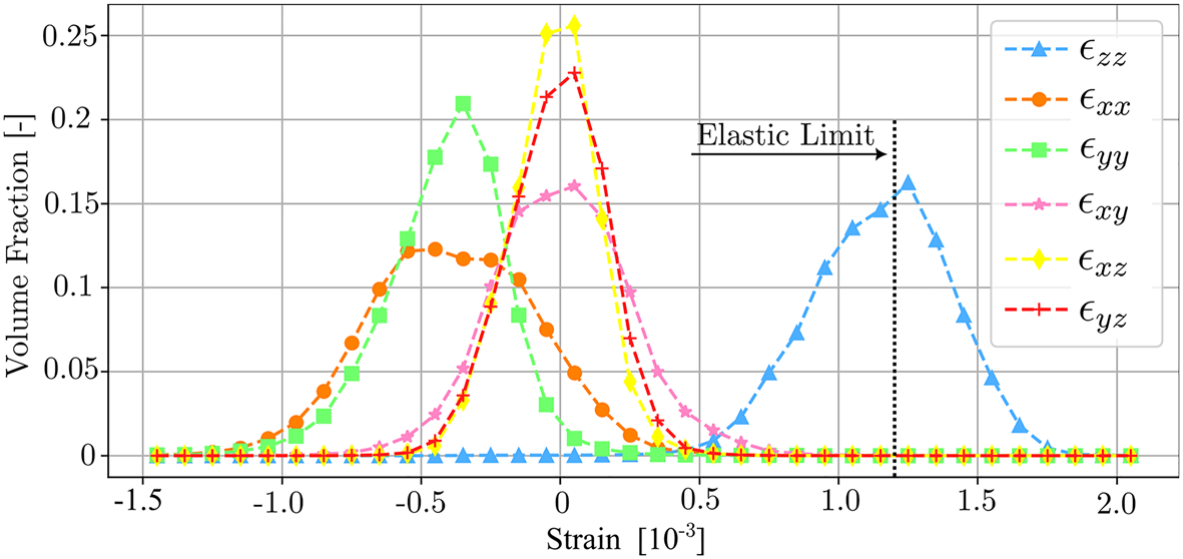
Distributions of the six independent components of the elastic strain tensor. Statistics over the 247,956 voxels in the sample volume. The axial strains along the tensile loading direction, $\epsilon _{zz}$, are distributed with a mean strain close to the elastic limit of the alloy.

We simultaneously reconstructed the elastic strain tensor field across the same volume (see figs. S8-S10). From these maps, we derived statistics on the distributions of intra-grain strain components, as illustrated in Fig. 4. The mean strain along the tensile loading axis (Fig. 4, $\epsilon _{zz}$) is 0.0011, to be compared to an elastic limit of 0.0012 (considering a proof stress of 85 MPa and an average tensile Young’s Modulus of 70 GPa). Remarkably, this implies that 42% of the volume is strained above the limit. The ratio between the mean transversal strains and the mean axial strain yields a Poisson ratio in the range 0.34-0.36, to be compared to a literature value of 0.33. Furthermore, the shear strain components $\epsilon _{xy}$, $\epsilon _{xz}$ and $\epsilon _{yz}$ have mean values of zero, as expected for uniaxial tension.

**Figure 5:**
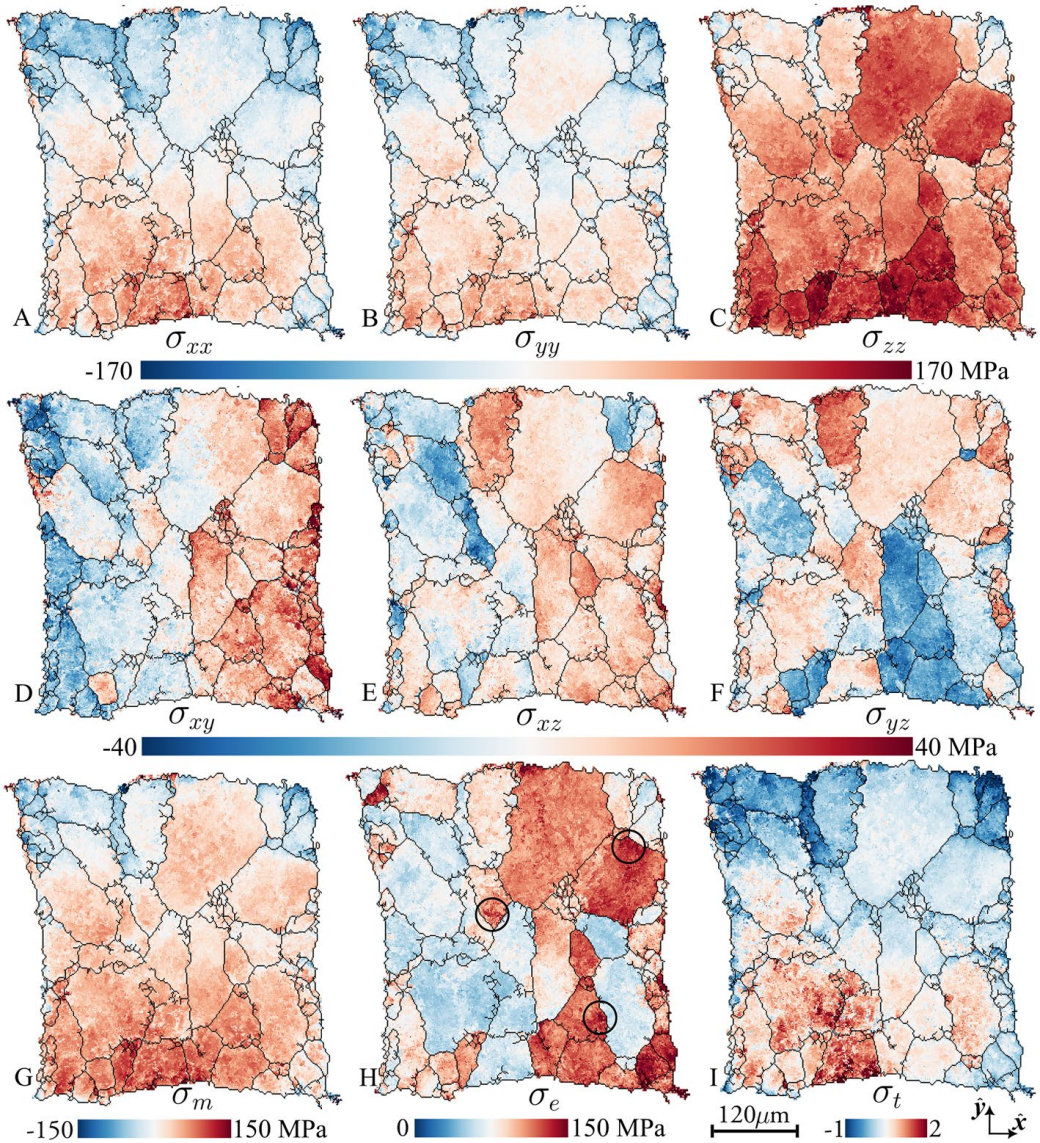
Reconstructed stress tensor field. (**A**–**C**) axial stress components $\varvec{\sigma }_{xx}$, $\varvec{\sigma }_{yy}$ and $\varvec{\sigma }_{zz}$, respectively. (**D**–**F**) Shear stress components $\varvec{\sigma }_{xy}$, $\varvec{\sigma }_{xz}$ and $\varvec{\sigma }_{yz}$, respectively. (**G**–**I**) Stress measures, derived from the stress tensor field: (**G**) the hydrostatic stress, $\varvec{\sigma }_{m}$, (**H**) the equivalent tensile stress, $\varvec{\sigma }_{e}$, and (**I**) the stress triaxiality, $\sigma _t = \sigma _m / \sigma _e$. Three local intra-grain stress concentrations have been marked by solid circles in (**H**). All maps relate to the central layer z2.

Using Hooke’s law^27^ and elastic constants for single crystal aluminum^45^, the strain field was converted to a corresponding stress field. The resulting maps of the stress components are shown in Fig. 5A–F (complemented by figs. S11-S13). Additionally, we present three derived measures. The local equivalent tensile stress, $\sigma _{e}$, Fig. 5H, is a measure of local distortion strain energy, commonly employed in predicting the onset of plastic deformation (also known as the Von Mises stress). Notably, this measure exceeds the true macroscopic flow stress at 32% elongation ($\sim 85$MPa) in $\sim$49% of the volume. The hydrostatic stress, $\sigma _{m}$, and the stress triaxiality, $\sigma _t = \sigma _m / \sigma _e$, shown in Fig. 5G,I, respectively, are crucial metrics^46^ in ductile failure theory. Elevated values tend to promote ductile failure through void expansion and coalescence. An analysis of the stress resolution based on out-of-balance forces is given in the supplementary materials (the analysis is based on the divergence of the stress field, see fig. S17). The standard deviations of the six ($\Delta \sigma _{xx}, \Delta \sigma _{yy}, \Delta \sigma _{zz}, \Delta \sigma _{xy}, \Delta \sigma _{xz}, \Delta \sigma _{yz}$) residual stress tensor components for each voxel were found to be in the range 8-12 MPa. This noise level is sufficient for testing most modeling approaches.

Inspection of Fig. 5 reveals a heterogeneous distribution of stress across various length scales, namely within the sample and at the inter-grain and the intra-grain levels. On the sample scale, the axial stress plots in Fig. 5A–C exhibit high values in the lower left corner, consequently resulting in high hydrostatic stress and triaxiality in Fig. 5G,I. This observation is attributed to the onset of necking in the sample, altering the shape of the initially square cross-section.

**Figure 6:**
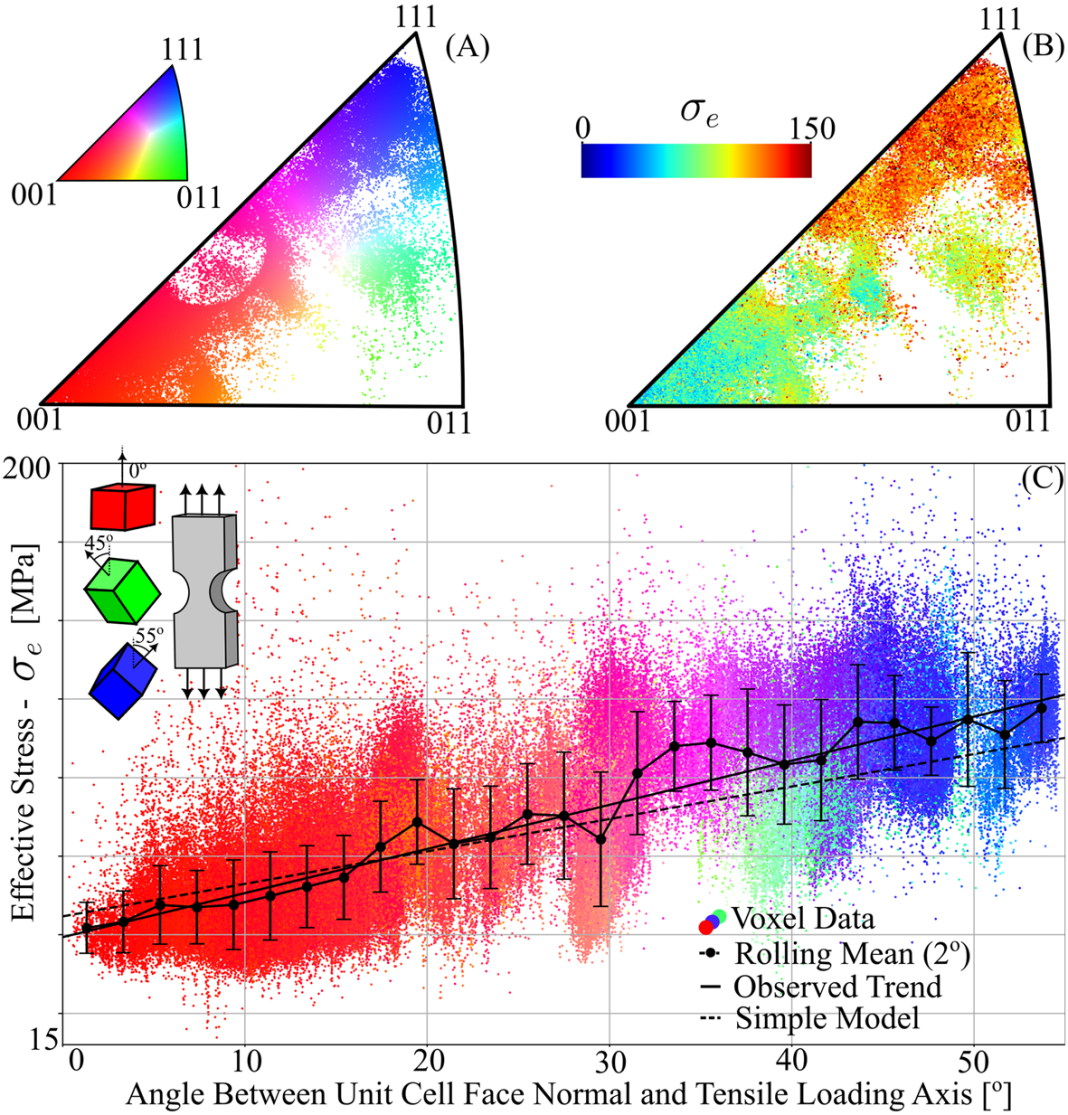
Orientation dependence of the effective stress. (**A**) IPF orientation distribution of the 247,956 voxels in the sample volume as viewed from the tensile loading axis. Fully saturated red corresponds to a local cubic unit cell with one of its six facets at a 90$^{\circ }$ angle to the loading axis, as illustrated in the top left corner of subfigure (**C**). (**B**) Corresponding local equivalent tensile stress, $\sigma _e$. (**C**) Relationship between $\sigma _e$ and the angle between the cubic unit cell faces and tensile loading axis. The scatter points are colored by the IPF color key in (**A**) and the error bars on the rolling mean curve mark one standard deviation. A linear least squares fit results in a slope of $\sim 1.4$ MPa / degree. In comparison, a simple model considering only the anisotropic elastic moduli of aluminum gives a slope of $\sim 1.0$ MPa / degree.

A distinction is also observed between the left and right parts of Fig. 5D–F,H. In particular, it is noteworthy that $\sigma _e$ primarily exceeds the macroscopic flow stress in the right part of the sample. A qualitative comparison with the crystallographic direction of the tensile axis in Fig. 3C reveals that grains featuring unit cell face normals aligned with the loading direction exhibit lower than average $\sigma _e$ values. Similar orientation effects of grain averaged stress right after the onset of plastic deformation have also been observed^47^. Comparing individual voxel orientations, using the inverse pole figure diagram in Fig. 6A, with the corresponding equivalent tensile stress (Fig. 6B) the coupling between stress and orientation state becomes evident. To quantify the influence of grain orientation on the stress state, we calculated the angle between the loading axis and the unit cell face normal for all voxels. A near linear relationship between this angle and the equivalent tensile stress (Fig. 6C, solid line) was observed. The positive trend can partly be explained by the anisotropy of aluminum, which exhibits a lower tensile stiffness for crystals that align one of their (cubic) unit cell face normals with the tensile axis. Uniformly randomly permuting all strain tensors in the sample spatially, while keeping the orientation field fixed, reveals the effect of this anisotropy and can be thought of as an expected stress trend had there been no correlation between orientation and strain states. The residual difference in slope between this expected trend (dashed line) and the actually observed trend (solid line) indicates systematic stress partitioning between (111) domains and (001) domains due to crystallography. This is attributed to the increased hardening of these orientations due to the higher plastic work required for elongating the grain. The clustering of these domains in the left and right part of the sample is the origin of the macroscopic partitioning, demonstrating the relevance of incorporating clusters of grains in modeling.

The linear trend of Fig. 6C predicts a stress fluctuation of 76 MPa over the sample volume. This stress variation is dominated by differences in inter-grain orientation and serves to measure the inter-grain stress heterogeneity in the sample (referred to as type II stress). In contrast, the distribution of equivalent tensile stress in Fig. 5H reveals stress variations below the scale of individual grains. These intra-grain stress variations, classified as type III stress, influence the extent of the error bars in Fig. 6C. To quantify the effective type III stress variation, we converted the error bars in Fig. 6C into equivalent full width half maxima. This conversion allowed us to establish an effective type III stress, yielding a value of 36 MPa. Consequently, our analysis indicates that the inter-grain stress range spans approximately twice the magnitude of the intra-grain stress range (76 MPa / 36 MPa). This finding underscores the importance of multi-scale modeling.

These three examples illustrate how local stress maps can be incorporated into damage and flow models, respectively. As another example, we propose that the scatter plot data presented in Fig. 6C can provide unique information for addressing the longstanding question regarding the significance of grain orientation versus grain interactions, and for quantifying intra-grain stress distributions as a function of grain orientation and the degree of deformation.

## Discussion

In our approach diffraction contrast imaging constitutes a spatial classification problem. In contrast to previous efforts which have generated orientation-strain maps based on a local maximum completeness criterion, our novel algorithm capitalizes on the concept of a multi-channel orientation-strain map encoding the set of possible solutions. By exploiting the spatial correlation in the orientation field we have devised a streamlined approach to reduce the vast solution set, that prioritizes fidelity to data while yielding a single stress-orientation field. The quality of the data is validated by our error analysis, figs. S14, S16-S17, and the correspondence with macroscopic properties, as shown e.g. in Fig. 4. Specifically, the error in spatial resolution was estimated to be less than the x-ray beam size, and the error in stress was found to range from 8 to 12 MPa^27^.

The experimental setup allows for simultaneous absorption contrast tomography, thereby establishing a direct link between microstructure and stress evolution and the nucleation and growth of voids and cracks. Our classification approach is scale-invariant and can be used in connection with S3DXRD studies of polycrystals featuring nano-meter sized grains, utilizing nano-beams as small as 100-250 nm^19,20,48^. By exploiting the out-of-plane spatial correlation in the orientating field the spatial resolution of our algorithm could be further enhanced. This upgrade can be implemented by expanding our 2D median filter to 3D, exploiting orientation correlations in subsequent $\varvec{\hat{z}}$-layers.

Furthermore, there exist several avenues for significantly enhancing strain and stress accuracy. Firstly, the advent of larger area detectors with more pixels will enable improved differentiation between spatial and strain degrees of freedom^49^. Secondly, our approach classifies the multi-channel orientation-strain map without using scattered intensity in the forward diffraction model. Including the intensities in the future is expected to yield substantial improvements in reconstruction quality. As an example, in 3DXRD, Monte-Carlo based algorithms using Gibbs priors have shown promise^40^.

From a broader perspective, we envision that X-ray diffraction microscopy can address a range of key questions in metallurgy by characterizing industrially relevant degrees of plastic deformation in 3D and in-situ. The capability to track deformation processes in three dimensions, spanning from the initial elastic regime through the plastic regime to component failure, will be pivotal moving forward. This capability holds the potential to bridge the gap between nano- and macroscale mechanics, addressing inquiries in predictive materials modeling on fracture phenomena such as creep, fatigue, and ductile failure theory. For instance, strain is known to induce grain boundary migration^50^, and recent results suggest that both inter- and intra-granular strain variations play key roles during grain growth^51^^52^. The class of 3D in-situ deformation data presented in this study are therefore key in validating and calibrating computer models aiming to simulate such phenomena^53–56^.

Furthermore, modern metalworking industries stand to benefit significantly from this advancement, enhancing their ongoing efforts to customize extrusion, machining, fabrication, and rolling processes. The optimization of these processes for material property enhancement offers sustainability and recycling advantages compared to alloying with additional chemical elements.

### Supplementary Information

Supplementary Information.

## Data Availability

All data, code, and materials used in the analysis will be provided to any researcher to reproduce or extend this analysis. The original synchrotron data and metadata is available at https://data.esrf.fr/doi/10.15151/ESRF-ES-578179478. The python codes used to analyze the data, the segmented diffraction peak files, and the processed results are available at https://zenodo.org/doi/10.5281/zenodo.11058847.
