# Supplementary material for: Microstructure and stress mapping in 3D at industrially relevant degrees of plastic deformation
Source: Sci Rep. 2024 Aug 30;14:20213. doi: 10.1038/s41598-024-71006-0 (PMC11364660; doi:10.1038/s41598-024-71006-0)
Supplement: Supplementary file 1 — Supplementary Information. [file 41598_2024_71006_MOESM1_ESM.pdf]

Supplementary Materials

1 Materials and Methods

1.1 Sample description

The sample material was AA1050, which was cold-rolled and subsequently recrystallized, resulting in an average grain size of 50-70μm and a typical recrystallization texture characterized by the predominance of the Cube orientation. This orientation signifies the alignment of the axes of the crystallographic unit cell with the rolling coordinate system.

The sample was cut such that the tensile axis was inclined at 45° to the rolling direction. Consequently, the specimen initially exhibited a prevalence of grains with the (011) plane normal aligned with the tensile axis and the (001) plane normal aligned with the X-ray beam direction.

The central part of the sample was notched with a radius of 1.55 mm and a square cross-section at the thinnest region measuring 0.24 mm^2 (see fig. S1). The total length of the notched region was 1.25 mm.

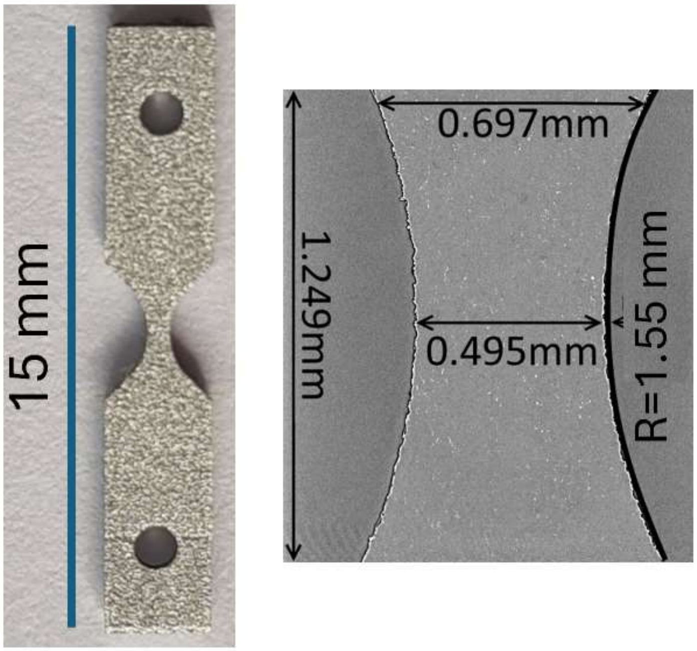

Figure S1: **Tensile specimen**. Left: Photo of the sample. Right: Tomographic slice of the central region. S3DXRD was conducted where the sample had the smallest cross-sectional area.

1.2 Experimental setup

Diffraction from the tensile specimen was imaged by scanning 3DXRD at the ID11 beamline of ESRF using the 3DXRD station at an X-ray energy of 42.5 keV. The sample was raster scanned in steps of 3μm by moving a $450 \times 450 \times 9\mu$ m^3 volume of interest across a 3 μm $\times$ 3 μm X-ray microbeam. At each scan position, the sample was rotated over 180°, and digital 2D (2K, 16-bit) images were acquired by integrating the diffraction signal over 1.0° intervals using a charge coupled device (CCD) FReLoN detector (*50*) placed 98.9 mm downstream of the sample with pixel dimensions of 47.2 $\times$ 47.2 μm^2. Following the density within the central part of the notched region of the sample was mapped by attenuation contrast imaging.

1.3 Stress rig

The sample was mounted in the Nanox stress rig, which was provided by the ESRF (*51*). Tensile deformation was then applied *in situ*. Both the engineering stress and the true stress (corrected for variable cross-sectional area) are shown in fig. S2. The S3DXRD study was conducted at approximately 65 MPa of engineering stress, which corresponds to the ultimate tensile strength of the alloy.

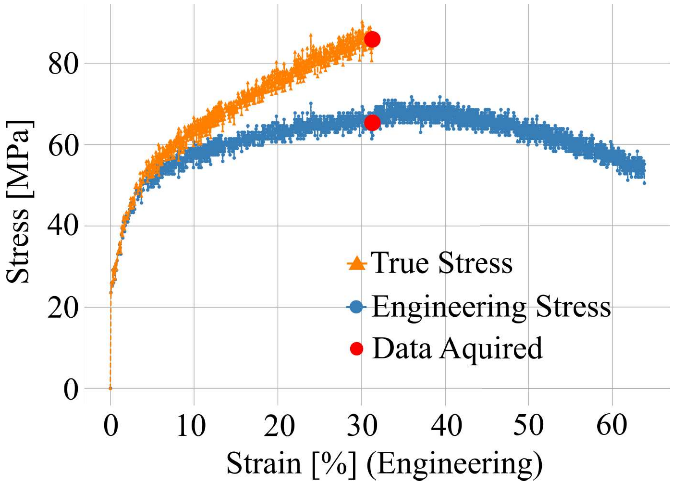

Figure S2: **Macroscopic stress-strain response**. The tensile stresses in the notched aluminium sample are plotted against tensile engineering strains. The point at which S3DXRD diffraction data was collected is indicated by a red dot.

Due to the sample shape, elongation is heterogeneous, and the local elongation at the location of the S3DXRD scans was determined to be 32% based on the reduction of the cross-sectional area (with consistent results provided by s3DXRD mapping and tomography). This result was also confirmed by tracking, in a similar sample, two pre-existing voids. The voids were initially situated 150 μm apart and located just below and above the S3DXRD region of interest. X-ray attenuation contrast tomography was used to track their position with deformation (results to be published elsewhere).

2 Methodology and data analysis

2.1 Orientation-strain map reconstruction algorithm

The data analysis pipeline used to reconstruct the voxelated orientation-strain map is delineated by the following six algorithmic steps (I-VI). Our algorithm was applied on a layer-by-layer basis, where each of the three layers (z1, z2, z3) was reconstructed independently. These reconstructed layers were then stacked to obtain a 3D reconstructed volume. The Python implementation of the following algorithm is openly available at `https://zenodo.org/doi/10.5281/zenodo.11058847`. While the computationally intensive parts of our algorithm were optimized for high-performance computing clusters, the code can be deployed on most CPU-based systems.

Step I - Peak segmentation

The diffraction patterns ($\sim 10^5$ 2K 16-bit images) underwent background correction, and the signal was segmented to produce sparse images where only intense signals (25 ADU) composed of connected pixels (with at least 9 pixels forming a spot) were retained. To label the connected pixels as diffraction spots we start by assigning each pixel to a gradient vector. The gradient vector of a pixel is here defined as the 2D vector that connects the pixel with its locally maximal neighbor pixel. Secondly, we assign unique labels to all pixels that constitute a local intensity maximum. Finally, we back-propagate the unique labels through the paths defined by the gradient vectors. Consequently, patches of connected pixels featuring multiple local maxima will be segmented into multiple distinct diffraction peaks. Using this method a list of $\sim$14,000,000 diffraction peaks were recorded, each associated with a detector position centroid, $\boldsymbol{r}$, a turntable rotation angle, ω, a sample translation, Δy, and an integrated intensity count.

Step II - Sample mask reconstruction

We utilized the summed diffraction signal on each collected detector frame to construct a sinogram, cf. fig. S3 A. The collected intensity was log-normalized per column in the sinogram, and the inverse radon transform (filtered back-projection) was applied. This process was implemented using a linear interpolation scheme and a standard ramp filter, as provided by the scipy library in Python (*52*).

The back-projected intensity for the central layer (z2) can be seen in fig. S3 B. The sample shape mask, depicted in black in fig. S3 C, was extracted from the back-projected reconstruction through the following steps: (I) thresholding the normalized reconstruction at 0.2, (II) selecting the largest connected domain in the thresholded reconstruction, and (III) filling all holes on the selected domain. The overlaid sample outline in red (fig. S3 C) was obtained by absorption tomography of the same layer in the sample.

Using a voxel size of $1.5 \times 1.5 \times 3\mu$ m^3, the number of voxels occupied by material was found to be 247,956. The advantages of selecting the x-y voxel dimensions smaller than the beam size are discussed elsewhere (*35, 36*).

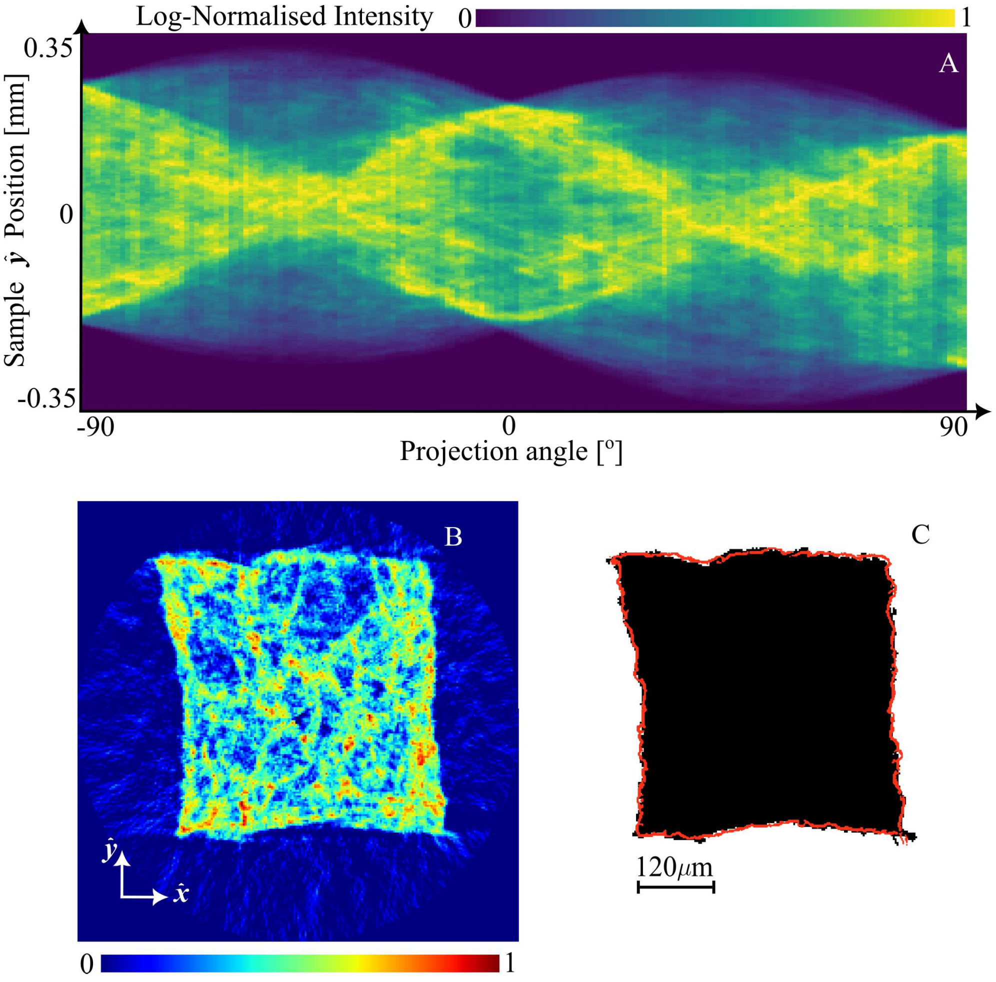

Figure S3: **Sample cross-section reconstruction**. The log-normalised sinogram representing the summed diffracted intensity (A) was subjected to filtered back-projection (B), facilitating the segmentation of the sample cross-section shape (C). The outline of the sample obtained independently from absorption tomography is highlighted in red. The figure corresponds to the central layer (z2).

Step III - Indexing

We indexed candidate crystal orientations at each voxel in the volume using ImageD11 (*28*) which implements indexing algorithms similar to those described in (*29–33*). By compiling a local diffraction data set composed of diffraction events where the distance between the X-ray beam and voxel centroid less than the beam size (3μm), candidate orientation-strain states were independently indexed for each voxel.

Explicitly, the voxel centroid position, x, y, given in a fixed laboratory coordinate system

(see (*21*) for coordinate system formalism) is a function of the turntable rotation angle, ω, as

$$x = x_0 \cos(\omega) - y_0 \sin(\omega)$$
$$y = x_0 \sin(\omega) + y_0 \cos(\omega) + \Delta y, \tag{1}$$

where (x_0, y_0) is the position of the voxel at $\omega = 0$ and Δy is the sample translation in y. The diffraction data associated with a specific voxel is defined to satisfy$|y| \leq 3\mu$m.

The sample diameter (0.24 mm) was sufficiently large to induce measurable shifts in the high-angle diffraction peak positions (with a sample-to-detector distance of 98.9 mm). Consequently, the diffraction data associated with the voxels were calibrated to account for the voxel position along the beam path (x). This entailed computing (elastic) scattered wave vectors, $\boldsymbol{k'}$, as

$$\boldsymbol{k'} = \frac{2\pi}{\lambda} \frac{\boldsymbol{r} - \boldsymbol{x}}{\sqrt{(\boldsymbol{r} - \boldsymbol{x})^T(\boldsymbol{r} - \boldsymbol{x})}}, \tag{2}$$

where $\boldsymbol{r}$ is the coordinate of a diffraction peak in 3D laboratory coordinates, and $\boldsymbol{x}$ is the voxel position according to equation (1). Diffraction vectors were defined as

$$\boldsymbol{G}_l = \boldsymbol{k'} - \boldsymbol{k}, \tag{3}$$

where $\boldsymbol{k}$ is the incident wave vector (which is parallel to $\hat{\boldsymbol{x}}$ in our setup). The corrected set of diffraction vectors, $\boldsymbol{G}_l^{(1)}, \boldsymbol{G}_l^{(2)}, ..., \boldsymbol{G}_l^{(m)}$, belonging to the voxel, was input to the indexer of ImageD11. The number of successfully indexed strain-orientation states found for each voxel depends on a minimum threshold of the number of peaks that are indexed within a given h, k, l error tolerance (see step IV and equation (8)).

With a very low h, k, l error tolerance the ImageD11 indexing algorithm may output many similar orientations, while a very high h, k, l error tolerance results in some peaks being assigned incorrectly. The values chosen here (0.03-0.05) correspond to an angular misorientation of 1-1.65 ° for the (111) ring and 0.3-0.5° for the (600) ring which corresponds to the highest angle measured. The (311) ring was found to be sufficient for initial indexing.

We relaxed the h, k, l error tolerance linearly from 0.03 to 0.05 until at least two distinct orientations could be indexed for the considered voxel. Similarly, the minimum number of diffraction peaks required to be indexed by an orientation was linearly relaxed from 216 to 108 alongside the h, k, l error tolerance. Additionally, the maximum number of candidate orientation-strain states allowed to be indexed by a single voxel was capped at 10.

Step IV - Orientation and strain refinement

We merged all diffraction peaks across detector frames, ensuring that each diffraction event could be associated to a unique set of Miller indices, h, k, l, along with a sample translation, Δy. Subsequently, for each voxel, we refined all candidate orientation matrices and strain tensors using all 13 available diffraction rings.

To describe the refinement procedure, we introduce the unit cell matrix, $(\boldsymbol{UB})^{-T}$, which contains the lattice unit cell vectors as its columns. The unit cell matrix is related to the diffraction vector, $\boldsymbol{G}_l$, according to the Laue equations

$$\boldsymbol{G}_l = \boldsymbol{\Omega} \boldsymbol{U} \boldsymbol{B} \boldsymbol{G}_{hkl}, \tag{4}$$

where $\boldsymbol{\Omega}$ is a rotation matrix describing the sample rotation with the turntable and $\boldsymbol{G}_{hkl}$ are integer Miller indices (c.f (*53*) for details on the diffraction formalism). For simplicity we refer to $\boldsymbol{G}$ in sample coordinates, such that

$$\boldsymbol{G} = \boldsymbol{U}\boldsymbol{B}\boldsymbol{G}_{hkl}. \tag{5}$$

In step III, we utilize equation (1) to allocate a sub-set of diffraction vectors, $\boldsymbol{G}^{(1)}, \boldsymbol{G}^{(2)}, ..., \boldsymbol{G}^{(m)}$, to the voxel under refinement. Since each diffraction vector is linked to both a turntable translation, Δy, and rotation, ω, the area of intersection between the X-ray beam and the voxel becomes variable. Furthermore, the beam profile deviates from a perfect top-hat function, exhibiting tails that extend beyond the nominal cross section of 3μm $\times$ 3μm. To accommodate these effects we introduce a data weight, w, which remains non-zero over a range extending beyond the nominal beam size. Importantly, the data weight should diminish with increasing distance between the X-ray beam and the centroid of the voxel. We selected the data weights as

$$w = \frac{1\mu\text{m}}{(y + \Delta y + 1\mu\text{m})}. \tag{6}$$

Consequently, when the voxel is centered on the beam ($y + \Delta y = 0$), we find $w = 1$, and when the voxel has a maximal offset from the beam ($y + \Delta y = 3\mu$m), we find $w = 0.25$.

To refine the unit cell matrix, $(\boldsymbol{U}\boldsymbol{B})^{-T}$, we introduce the data vector

$$\bar{\boldsymbol{G}} = \begin{bmatrix} \boldsymbol{G}^{(1)} \\ \boldsymbol{G}^{(2)} \\ \vdots \\ \boldsymbol{G}^{(m)} \end{bmatrix} \tag{7}$$

where $\boldsymbol{G}^{(i)}$ are diffraction vectors indexed by the candidate unit cell matrix, $(\boldsymbol{U}\boldsymbol{B})^{-T}$, adhering to the h, k, l error tolerance, $e_{hkl} = 0.05$. Explicitly, the data satisfying

$$||(\boldsymbol{U}\boldsymbol{B})^{-1}\boldsymbol{G}^{(i)}||_2 < e_{hkl} \tag{8}$$

was considered, where $||\cdot||_2$ denotes the Euclidean norm. Furthermore, we introduce a flattened format of the unit cell matrix as

$$\boldsymbol{\rho} = \begin{bmatrix} UB_{11} \\ UB_{12} \\ UB_{13} \\ UB_{21} \\ UB_{23} \\ UB_{23} \\ UB_{31} \\ UB_{32} \\ UB_{33} \end{bmatrix} \tag{9}$$

allowing us to define a system matrix, $\boldsymbol{H}$, populated by the integer Miller indices, h, k, l, such that

$$\bar{\boldsymbol{G}} = \boldsymbol{H}\boldsymbol{\rho}. \tag{10}$$

We can now perform the least squares fit

$$\boldsymbol{\rho} = (\boldsymbol{H}^T\boldsymbol{W}^T\boldsymbol{W}\boldsymbol{H})^{-1}\boldsymbol{H}^T\boldsymbol{W}^T\boldsymbol{W}\bar{\boldsymbol{G}} \tag{11}$$

where $\boldsymbol{W}$ is a diagonal weight matrix according to equation (6).

The angular shift in a diffraction peak centroid can be attributed to a directional strain acting across the diffracting lattice planes (*54*) (*55*). Therefore, the precision in the elastic strain tensor ($\boldsymbol{\epsilon}$) fit can be further enhanced by directly exploiting these angular shifts in the diffraction data. Specifically, following (*56*) each diffraction peak centroid was converted into a single scalar measurement of directional strain, ε, as

$$\varepsilon = \frac{\boldsymbol{G}^T\boldsymbol{G}_0}{\boldsymbol{G}^T\boldsymbol{G}} - 1, \tag{12}$$

where $\boldsymbol{G}$ is the measured diffraction vector and $\boldsymbol{G}_0$ is a model diffraction vector. The model diffraction vector is computed using the local fitted orientation matrix, $\boldsymbol{U}$, and an undeformed reference unit cell, which is defined based on tabulated values ($a = 4.04$Å, $\alpha = 90.0°$) (*57*).

Considering the symmetric elastic strain tensor, $\boldsymbol{\epsilon}$, as the unknown parameter we defined the model equation

$$\varepsilon = \frac{\boldsymbol{G}^T\boldsymbol{\epsilon}\boldsymbol{G}}{\boldsymbol{G}^T\boldsymbol{G}}, \tag{13}$$

which represents a strain tensor measurement in the direction normal to the lattice planes associated with $\boldsymbol{G}$. Many such measurement form a linear set of equations

$$\boldsymbol{y} = \boldsymbol{M}\boldsymbol{s}, \tag{14}$$

where $\boldsymbol{s}$ holds the six unknown strain tensor components

$$\boldsymbol{y} = \begin{bmatrix} \varepsilon_{xx} \\ \varepsilon_{yy} \\ \varepsilon_{zz} \\ \varepsilon_{xy} \\ \varepsilon_{xz} \\ \varepsilon_{yz} \end{bmatrix}, \tag{15}$$

and the rows of the system matrix, $\boldsymbol{M}$, are defined as

$$M_j = \begin{bmatrix} \kappa_1^2 & \kappa_2^2 & \kappa_3^2 & 2\kappa_1\kappa_2 & 2\kappa_1\kappa_3 & 2\kappa_2\kappa_3, \end{bmatrix} \tag{16}$$

and

$$\boldsymbol{\kappa} = \begin{bmatrix} \kappa_1 \\ \kappa_2 \\ \kappa_3 \end{bmatrix} = \frac{\boldsymbol{G}}{||\boldsymbol{G}||_2}. \tag{17}$$

Assuming zero-mean isotropic Gaussian noise in $\boldsymbol{G}$, with standard deviation $\sigma_g = 10^{-4}$, we introduce the approximate weights

$$w = \left(\frac{1\mu\text{m}}{(y + \Delta y + \mu\text{m})}\right)\left(\sigma_g^2\frac{\boldsymbol{G}_0^T\boldsymbol{G}_0}{\boldsymbol{G}^T\boldsymbol{G}}\right)^{-1/2}, \tag{18}$$

and define outliers as

$$\varepsilon > \mu_\varepsilon + 3.5\sigma_\varepsilon \quad \text{or} \quad \varepsilon < \mu_\varepsilon - 3.5\sigma_\varepsilon, \tag{19}$$

where μ_ε and σ_ε are the mean and standard deviation of the measured directional strains, respectively.

The refined strain tensor was determined using the weighted least squares solution

$$\boldsymbol{s} = (\boldsymbol{M}^T\boldsymbol{W}^T\boldsymbol{W}\boldsymbol{M})^{-1}\boldsymbol{M}^T\boldsymbol{W}^T\boldsymbol{W}\boldsymbol{y}. \tag{20}$$

where $\boldsymbol{W}$ is a diagonal weight matrix according to equation (18), and outliers have been disregarded according to equation (19).

This summarises our refinement step for the strain and orientation. The outlined procedure was executed independently for each voxel and each candidate orientation in the indexed volume.

Step V - Spatial filtering

Combining the refined strain-orientation candidates with the sample mask resulted in a $\sim 290 \times 290 \times 3$ voxel volume with a list of candidate grain orientations and strain tensors attached to each voxel. This multi-channel orientation-strain map represents a vast set of possible solutions to an inversion problem. On average, each voxel held 6 orientation channels, resulting in a huge number of possible solutions by randomly permuting choices. This complexity makes any approaches relying on trial-and-error selection computationally impossible.

The next step of our reconstruction approach aims to establish a single-channel orientation-strain map by selecting one solution from the multi-channel orientation-strain map. It is possible to use the maximum number of unique diffraction peaks (maximum completeness) as a selection criterion (*23, 34, 43, 44*). However, due to the challenges of diffraction spot overlap, we found this criterion alone to result in noisy orientation-strain maps with artifact orientations scattered over the reconstructed volume. To overcome this obstacle, we aimed to preserve spatial correlation in the final orientation-strain map while maximizing the number of unique diffraction peaks in the reconstruction. This step in our reconstruction approach is summarized by the following 5 actions:

1. We selected an initial (noisy) single-channel orientation-strain map by assigning, for each voxel, the candidate orientation that indexed the most (unique) diffraction peaks.

2. We converted the noisy single-channel orientation-strain map into a color image (one RGB value per voxel) using an inverse pole figure color map.

3. We ran a 6×6 median filter across each color channel (Red, Green, Blue) in the image, resulting in a new, artificial color image with substantially reduced noise.

4. We converted each channel in the original multi-channel map into RGB values again using the inverse pole figure color map.

5. We selected a new single-channel orientation-strain map by comparing our artificial color image to the RBG values of the multi-channel map. Each voxel in the new single-channel map was assigned the candidate orientation-strain state from the multi-channel map that featured the smallest Euclidean norm between RGB tuples.

Step VI - Diffraction centroid calibration

In Step III of our analysis, we approximated diffraction to originate from the centroids of the individual voxels in the sample. This approximation can be improved by recognising that each diffraction peak is associated to a spatially extended domain in the sample. These diffracting domains can be approximated as intersections between the X-ray beam and the voxel grid, considering only voxels that fulfil the Laue equations (5). Using the orientation-strain map obtained from step V, it is therefore possible to compute an updated approximation of the diffraction origins ($\boldsymbol{x}$). For each observed diffraction peak, we computed the centroid of the corresponding diffracting domain in the sample and inserted the result into equation (2), effectively correcting the diffraction vector data set. The analysis in step IV was then rerun, updating the multi-channel orientation-strain map using the corrected data, and the new multi-channel map was input to step V resulting in a final reconstructed single-channel orientation-strain map.

Algorithm summary

We indexed orientation-strain states on a voxel grid, allowing for multiple indexed candidates per voxel. Subsequently, we refined the indexed multi-channel orientation-strain map based on voxel positions and used a median filtering operation to select an approximate solution. Using the approximate solution, we corrected our diffraction data and reran our refinement analysis and filtering algorithm. The final result is a voxelated single-channel orientation-strain map.

Our approach ensures that the final reconstruction is among the set of possible indexed solutions, adhering to diffraction data, while simultaneously suppressing noise by leveraging the assumption of spatial correlation among local crystal orientations. It's worth noting that the median filtering operations are conducted with a filter size much smaller than the typical grain size, ensuring the ability to resolve spatial variations across the grains. However, strain and orientation variations localised on an even smaller scale, such as in the vicinity of boundaries, may be subject to larger errors, as discussed in section 3.2.

It is also worth to mention that since the sample cross-section is relatively large compared with the detector-to-sample distance (0.24mm respectively 98.9mm), our correction for the spatial origin of diffraction not only suppresses spurious strain gradients that arise due to the finite sample size but also help to select the correct grain orientation locally during indexing (step III).

2.2 Stress conversion

We converted the strain tensor fields to stress tensor fields, voxel by voxel, using the elastic constants of Aluminum, $D_{11} = 104$ GPa, $D_{12} = 73$ GPa, and $D_{44} = 32$ GPa (*45*). Since the material parameters are given in relation to the IEEE standard (*58*), conversion from strain to stress must take place in a local crystal coordinate system that aligns the Cartesian axes with the cubic unit cell axes. Explicitly, the fourth order elasticity tensor, given in the crystal reference frame, was taken as

$$D = \begin{bmatrix} 104 & 73 & 73 & 0 & 0 & 0 \\ 73 & 104 & 73 & 0 & 0 & 0 \\ 73 & 73 & 104 & 0 & 0 & 0 \\ 0 & 0 & 0 & 32 & 0 & 0 \\ 0 & 0 & 0 & 0 & 32 & 0 \\ 0 & 0 & 0 & 0 & 0 & 32 \end{bmatrix} \text{GPa}. \tag{21}$$

The strain tensor was converted to crystal coordinates as

$$\boldsymbol{\epsilon}^{(c)} = \boldsymbol{U}^T \boldsymbol{\epsilon} \boldsymbol{U}, \tag{22}$$

using the local orientation matrix, $\boldsymbol{U}$, associated to the voxel. The corresponding crystal coordinate stress components in the considered voxel could then be computed from Hooke's law as

$$\begin{bmatrix} \sigma_{xx}^{(c)} \\ \sigma_{yy}^{(c)} \\ \sigma_{zz}^{(c)} \\ \sigma_{yz}^{(c)} \\ \sigma_{xz}^{(c)} \\ \sigma_{xy}^{(c)} \end{bmatrix} = \begin{bmatrix} 104 & 73 & 73 & 0 & 0 & 0 \\ 73 & 104 & 73 & 0 & 0 & 0 \\ 73 & 73 & 104 & 0 & 0 & 0 \\ 0 & 0 & 0 & 32 & 0 & 0 \\ 0 & 0 & 0 & 0 & 32 & 0 \\ 0 & 0 & 0 & 0 & 0 & 32 \end{bmatrix} \begin{bmatrix} \epsilon_{xx}^{(c)} \\ \epsilon_{yy}^{(c)} \\ \epsilon_{zz}^{(c)} \\ 2\epsilon_{yz}^{(c)} \\ 2\epsilon_{xz}^{(c)} \\ 2\epsilon_{xy}^{(c)} \end{bmatrix} \text{GPa}, \tag{23}$$

where superscript (c) denotes crystal reference frame. Conversion back to sample coordinates was then performed as

$$\boldsymbol{\sigma} = \boldsymbol{U} \boldsymbol{\sigma}^{(c)} \boldsymbol{U}^T. \tag{24}$$

Note that for materials that do not exhibit the cubic symmetry the above conversion between sample and crystal frame should feature an additional matrix transformation to ensure consistency with the IEEE conventions.

For completeness, we provide explicitly the definitions for the equivalent tensile stress

$$\sigma_e = \sqrt{3J_2}, \tag{25}$$

where the second stress invariant, J_2, is defined as

$$J_2 = \sqrt{\frac{1}{2} \sum_i \sum_j s_{ij} s_{ji}}, \tag{26}$$

and the deviatoric stress, s_{ij}, is given by

$$s_{ij} = \sigma_{ij} - \frac{1}{3} \sigma_m \delta_{ij}, \tag{27}$$

where δ_{ij} is Kronecker's delta, and the hydrostatic stress, σ_m, is defined as

$$\sigma_m = \frac{1}{3} (\sigma_{11} + \sigma_{22} + \sigma_{33}). \tag{28}$$

Alphabetical and numerical subscripts for strain and stress tensors are equivalent, given by

$$\boldsymbol{\sigma} = \begin{bmatrix} \sigma_{11} & \sigma_{12} & \sigma_{13} \\ \sigma_{21} & \sigma_{22} & \sigma_{23} \\ \sigma_{31} & \sigma_{32} & \sigma_{33} \end{bmatrix} = \begin{bmatrix} \sigma_{xx} & \sigma_{xy} & \sigma_{xz} \\ \sigma_{yx} & \sigma_{yy} & \sigma_{yz} \\ \sigma_{zx} & \sigma_{zy} & \sigma_{zz} \end{bmatrix}. \tag{29}$$

2.3 KAM filter & Grain Boundary Identification

Within each slice in z, Kernel average misorientations (KAM) (*59*) were computed, considering a neighborhood defined by the beam-size (3μm) and a lower misorientation threshold of 1.8^o (fig. S4). Specifically, the neighborhood matrix was taken as

$$\begin{bmatrix} 0 & 0 & 1 & 0 & 0 \\ 0 & 1 & 1 & 1 & 0 \\ 1 & 1 & 1 & 1 & 1 \\ 0 & 1 & 1 & 1 & 0 \\ 0 & 0 & 1 & 0 & 0 \end{bmatrix}. \tag{30}$$

The resulting KAM maps are shown in fig. S4. Grain and sub-grain boundaries were identified for each z-layer separately by:

1. Binarising the KAM map at a threshold of 4°.
2. Applying a morphological thinning to the binary map.
3. Pruning the binary skeleton of small residual objects (min feature size 64 pixels with a 2-pixel connectivity).

The resulting binary grain boundary skeletons are presented in fig. S5.

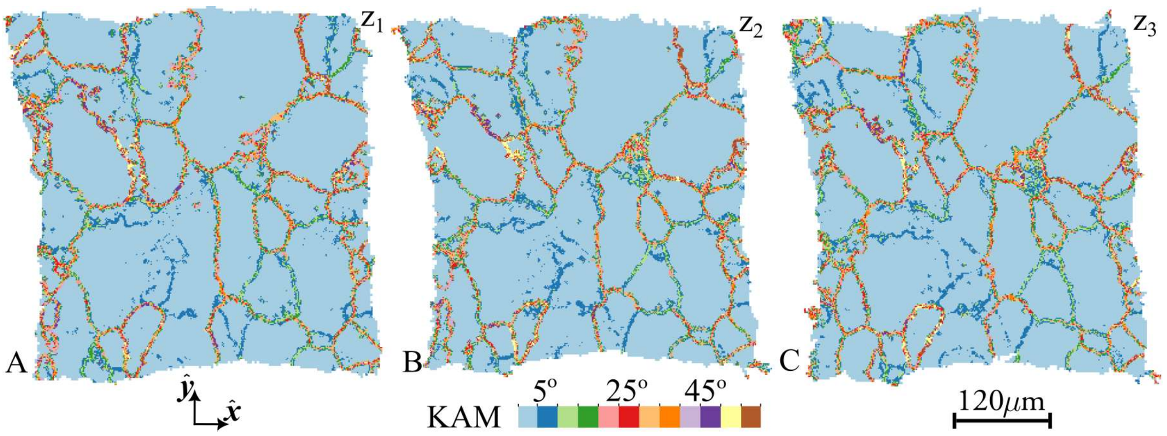

Figure S4: **KAM maps** for the three reconstructed z-layers, z1,z2 and z3 (A,B,C, respectively).

2.4 Grain orientations and intra-grain orientation distributions

For each z-layer, all connected component regions found from the analysis in section 2.3, containing more than 25 voxels, were identified as grains, and the mean unit cell over each such grain was computed. The resulting average orientations (each associated to one z-slice of one segmented grain) are plotted as inverse pole figures in fig. S6. The total number of represented grain z-slices is 179. As seen in fig. S6, this procedure revealed a typical tensile texture where

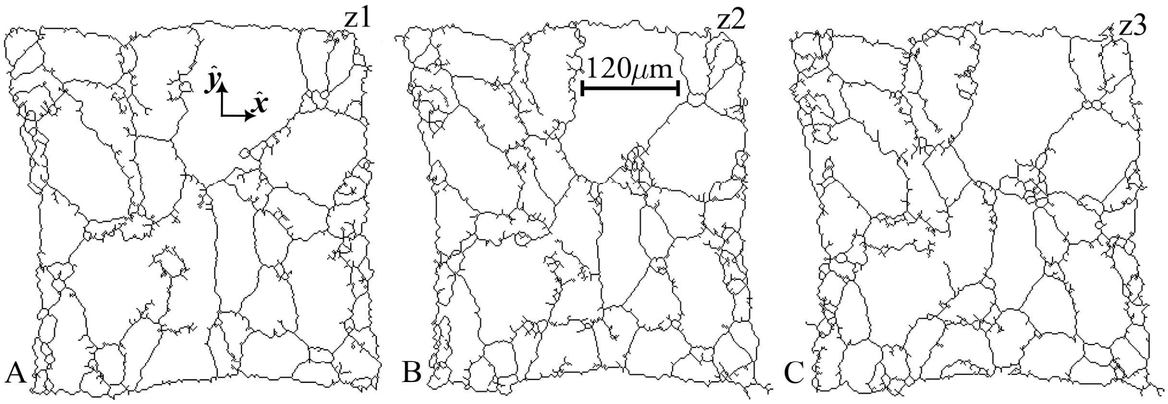

Figure S5: **Grain boundary skeletons** obtained from the KAM maps in fig. S4.

the crystallographic direction aligned with the tensile axis (z-axis) spans the (001)-(111) line of the triangle for most grains, and grains with tensile direction along (011) are scarce.

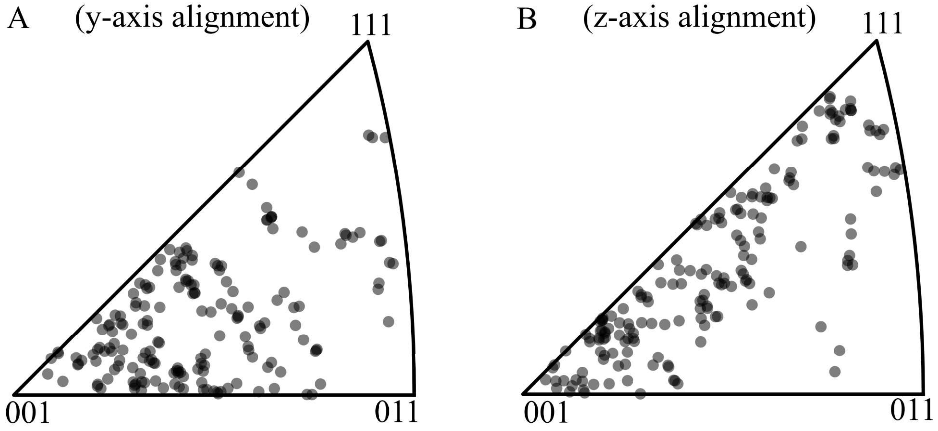

Figure S6: **Macroscopic texture**. Inverse pole figures of grain mean orientations with respect to (A) y-axis alignment and (B) z-axis alignment. The lattice plane normal to the 011 family is seen to rarely align with the z-axis (which is also the tensile axis), as is typical after tensile deformation. Each point in the inverse pole figure corresponds to the mean orientation of a segmented x-y grain-slice.

The Intra-grain misorientation distributions around the grain average orientation showed significant broadening, as shown in fig. S7. Particularly, elevated levels of misorientation above 10 ^{o} were observed in two large grains that featured multiple low-angle sub-grain boundaries (fig. S7 grains b and e). The presence of orientation gradients in the orientation map are indicators of plastic strain, characterised by slipping of crystal planes, causing the lattice to warp, which subsequently resulted in severe diffraction peak arcing and diffraction peak overlap.

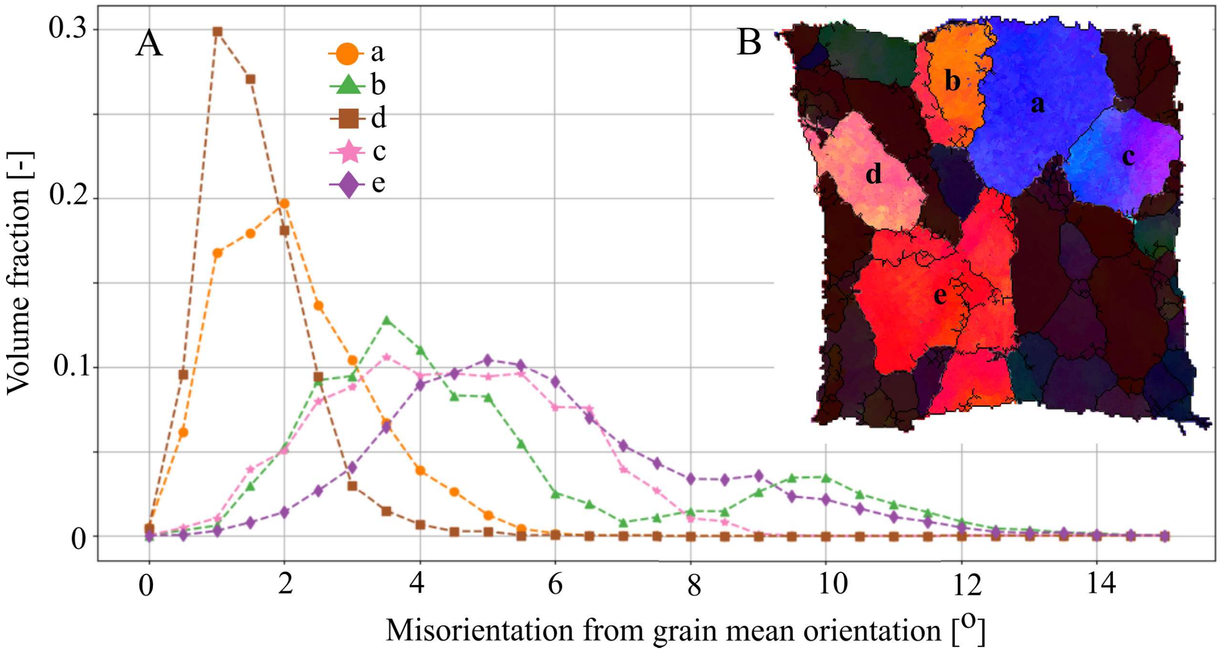

Figure S7: **Intra-grain misorientations**. Histograms of misorientation (A) for the five largest grains in the central layer (z2) of the sample (B, a-e). Each histogram (a-e) shows misorientations in relation to the grain mean orientation. Grain volumes were segmented using a flood fill approach, searching for spatially connected regions with local misorientations of less than 4.0°.

2.5 Strain Tensor Maps

The full strain tensor field, reconstructed using the algorithm described in section 2.1, is presented in figs. S8-S10. The three figures illustrates the three separate layers, z1, z2 and z3.

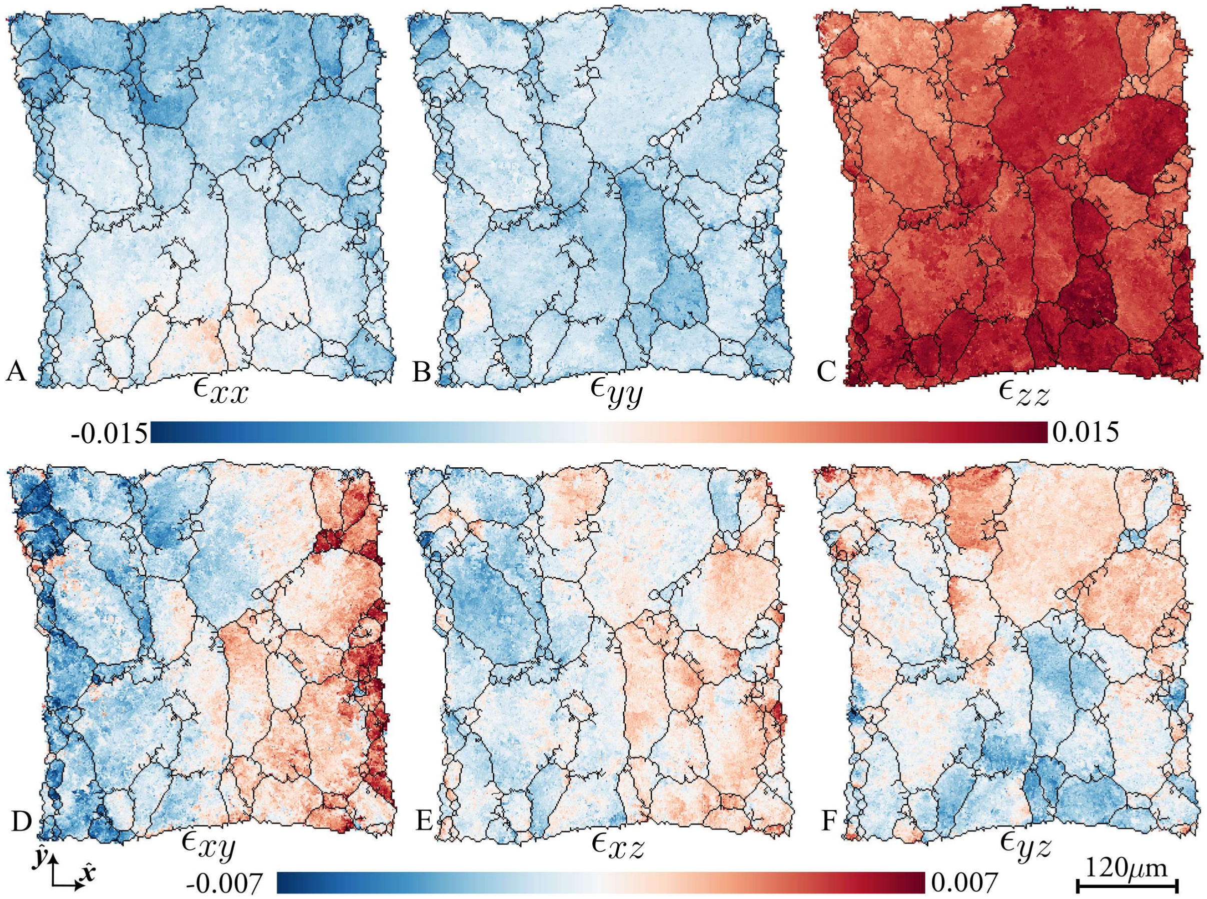

Figure S8: **Reconstructed strain tensor field in bottom (z1) layer**. Axial strain, ϵ_{xx}, ϵ_{yy}, ϵ_{zz} (A-C), and shear strain, ϵ_{xy}, ϵ_{xz}, ϵ_{yz} (D-F).

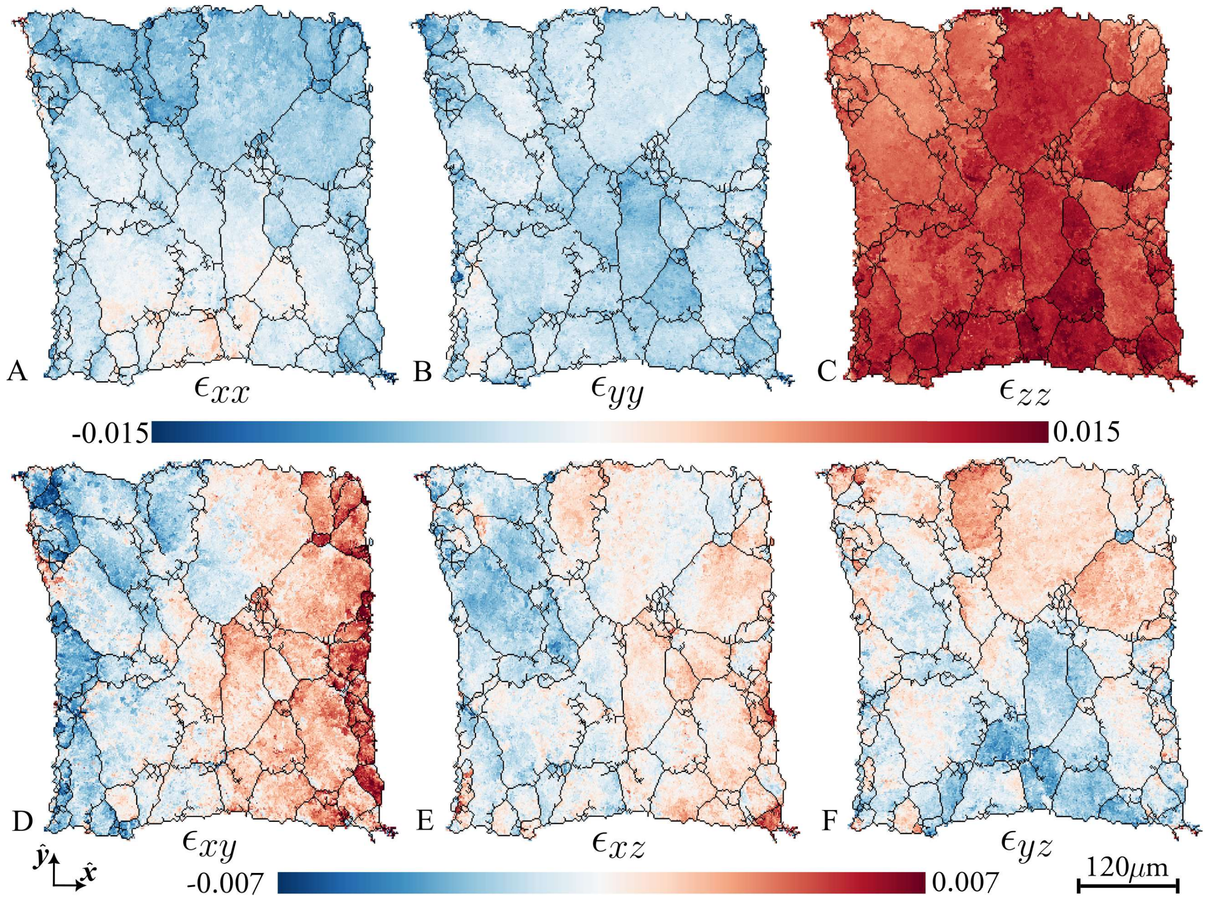

Figure S9: **Reconstructed strain tensor field in central (z2) layer**. Axial strain, ϵ_{xx}, ϵ_{yy}, ϵ_{zz} (A-C), and shear strain, ϵ_{xy}, ϵ_{xz}, ϵ_{yz} (D-F).

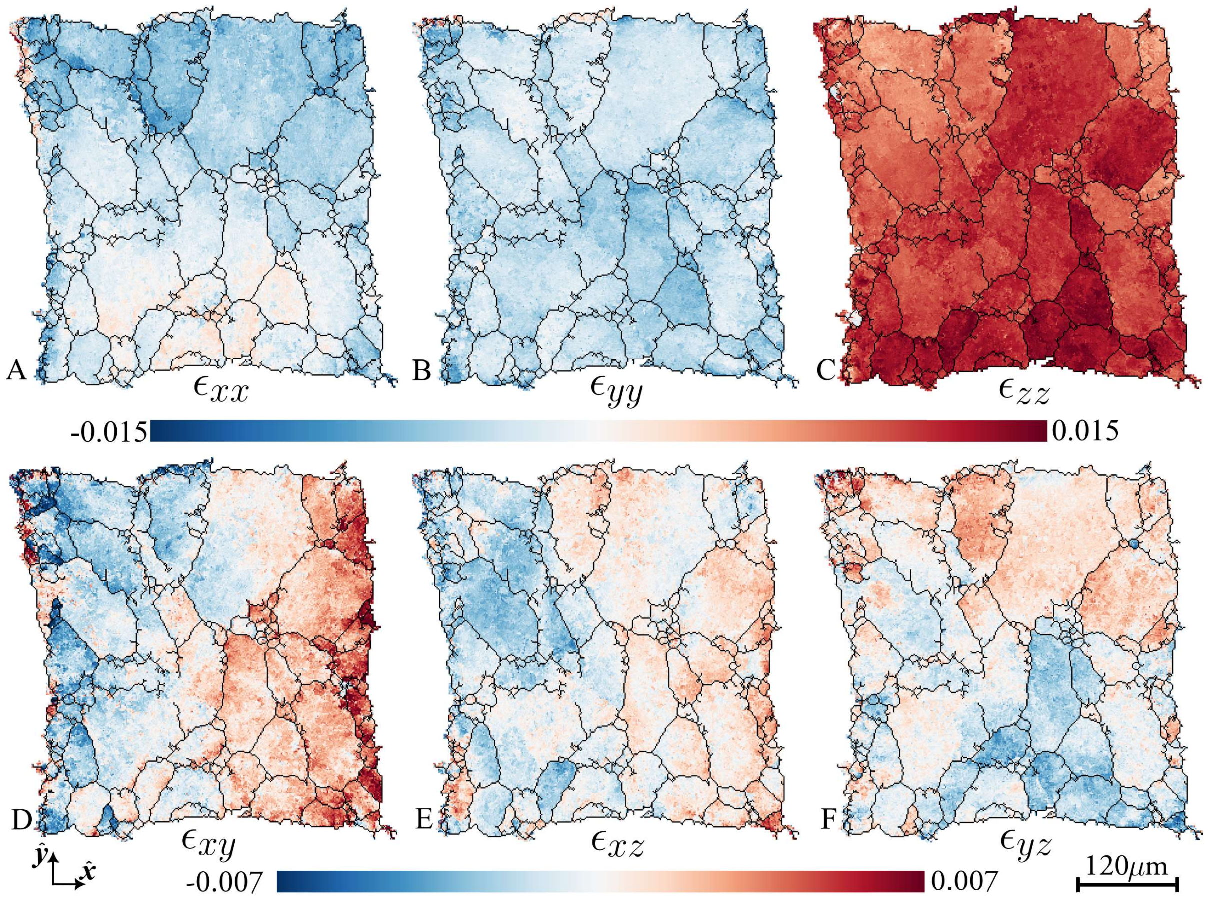

Figure S10: **Reconstructed strain tensor field in top (z3) layer**. Axial strain, ϵ_{xx}, ϵ_{yy}, ϵ_{zz} (A-C), and shear strain, ϵ_{xy}, ϵ_{xz}, ϵ_{yz} (D-F).

2.6 Stress Tensor Maps

The full stress tensor field, computed from the strain tensor fields in figs. S8-S10, are shown as figs. S11-S13. These complements the main material of the paper by showcasing the stress tensor for all three layers, z1, z2 and z3.

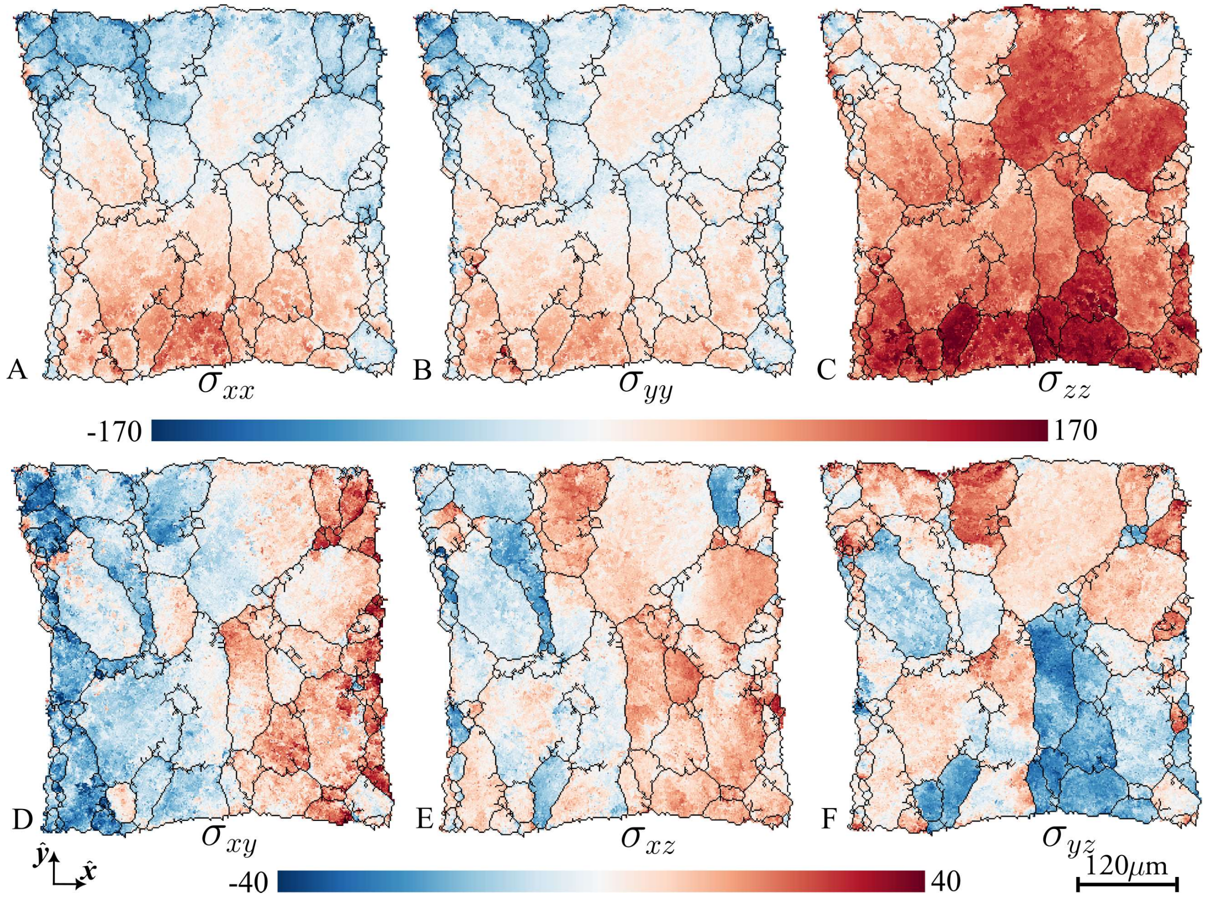

Figure S11: **Reconstructed stress tensor field in bottom (z1) layer**. Axial stress, σ_{xx}, σ_{yy}, σ_{zz} (A-C), and shear stress, σ_{xy}, σ_{xz}, σ_{yz} (D-F), stress in the bottom (z1) layer.

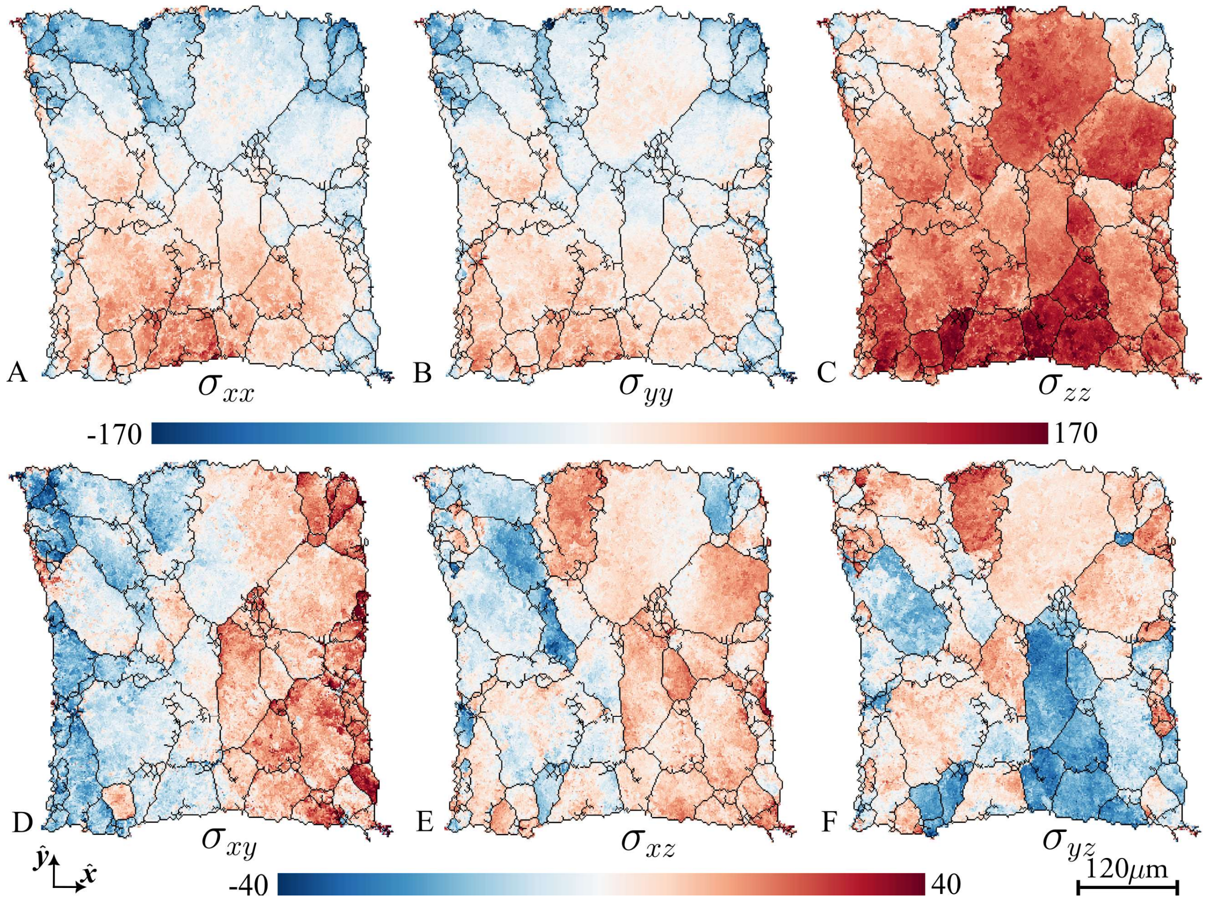

Figure S12: **Reconstructed stress tensor field in central (z2) layer**. Axial stress, σ_{xx}, σ_{yy}, σ_{zz} (A-C), and shear stress, σ_{xy}, σ_{xz}, σ_{yz} (D-F). This figure reproduces parts of the results in fig. 4 of the main paper.

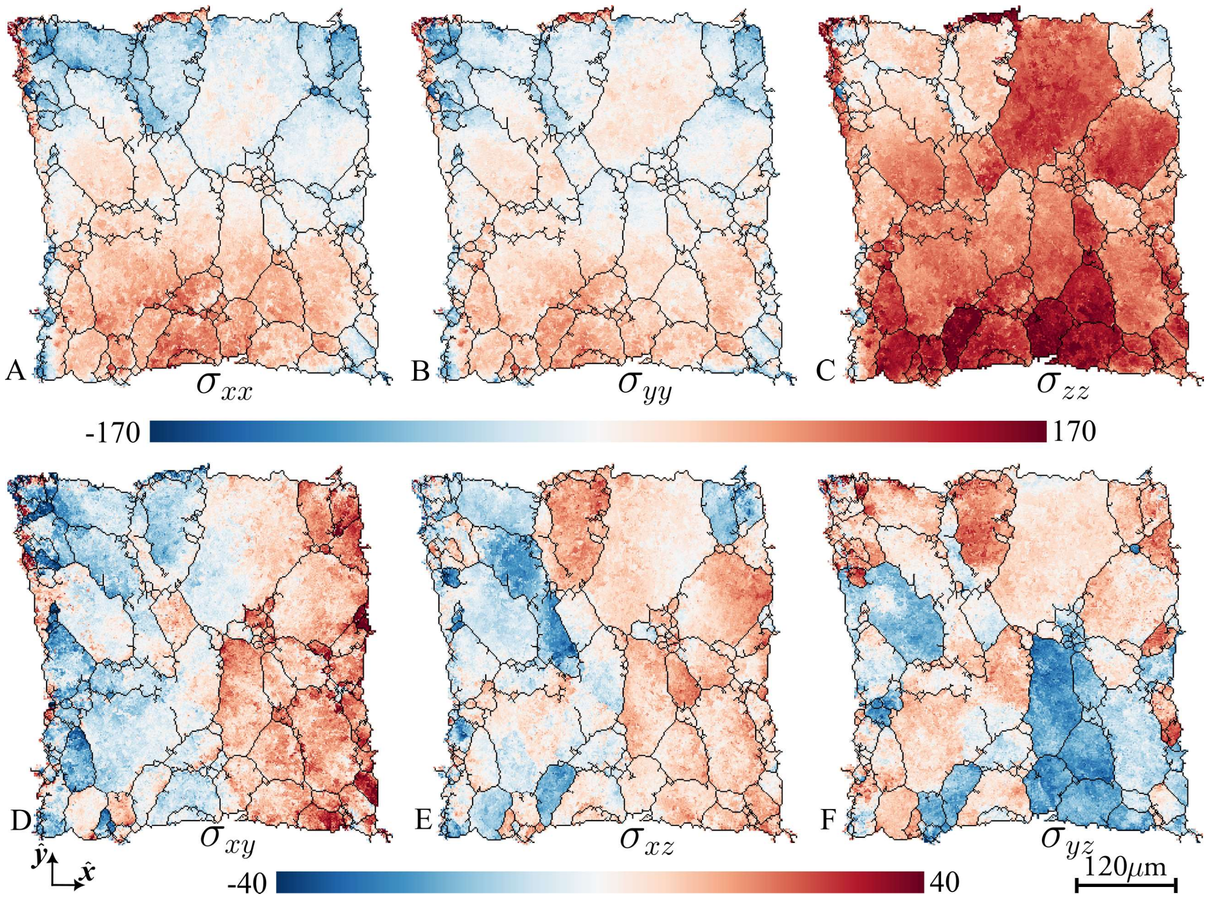

Figure S13: **Reconstructed stress tensor field in top (z3) layer**. Axial stress, σ_{xx}, σ_{yy}, σ_{zz} (A-C), and shear stress, σ_{xy}, σ_{xz}, σ_{yz} (D-F), .

3 Error Estimation

3.1 Spatial Resolution

To estimate the spatial resolution in our reconstruction, we utilised the displacements of grain boundaries between consecutive z-layers in the grain volume. These displacements arise from a combination of the actual change in grain shape and an unknown spatial reconstruction error. Assuming that these two effects are uncorrelated and that the distribution of error displacements at grain boundaries follow a multivariate Gaussian distribution, we could infer the spatial resolution by comparing the reconstructed grain boundary skeletons of consecutive z-layers. We found an error standard deviation close to the voxel size ($\sim$1.5μm). For further details and the statistical characteristics of the error, refer to table S1. Profiles illustrating the estimated grain boundary error displacement distribution are presented in fig. S14. Below, we provide a comprehensive explanation of the methodology employed to obtain this estimate.

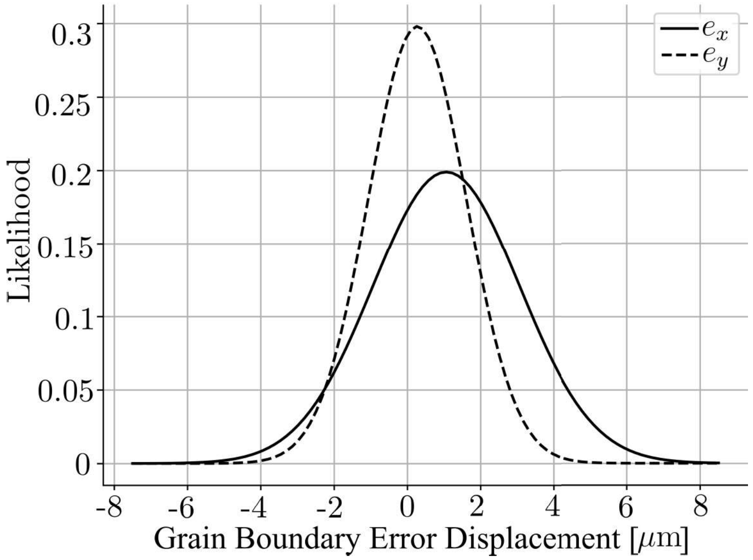

Figure S14: **Estimated spatial reconstruction error**. The likelihood of error displacements at grain boundaries in the reconstruction is shown. The error standard deviation is close to the voxel size ($\sim$1.5μm).

Assume $\boldsymbol{p}$ denotes a point on a grain boundary and let $\hat{\boldsymbol{n}}$ be the associated normal to the grain boundary tangent plane at $\boldsymbol{p}$. Given an increment, Δz, in the direction of $\hat{\boldsymbol{z}}$, a point $\boldsymbol{p} + \Delta \boldsymbol{p}$, is reached by following the tangent plane with normal $\hat{\boldsymbol{n}}$ starting from $\boldsymbol{p}$. The neighbouring grain boundary point $\boldsymbol{p} + \Delta \boldsymbol{p}$ is here defined as the closest point (in a Euclidean sense) to $\boldsymbol{p}$ at $z = p_z + \Delta z$. The situation is geometrically depicted in fig. S15.

Metric	Value
Standard deviation in x	$2.01\ \mu$m
Standard deviation in y	$1.34\ \mu$m
Mean	$\left[1.06, 0.27\right]^T\ \mu$m
Covariance	$\begin{bmatrix} 4.02 & 0.57 \\ 0.57 & 1.79 \end{bmatrix}\ \mu\text{m}^2$

Table S1: Statistics of estimated grain boundary displacement error distribution.

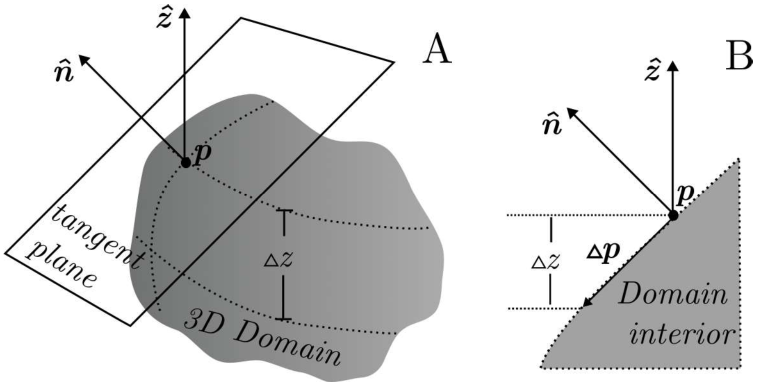

Figure S15: **Grain boundary curvature postulate**. A 3D domain has a tangent plane at $\boldsymbol{p}$ with normal $\hat{\boldsymbol{n}}$ (A). For an increment Δz along $\hat{\boldsymbol{z}}$ the closest point to $\boldsymbol{p}$ on the domain boundary is $\boldsymbol{p} + \Delta\boldsymbol{p}$ (B).

Projecting $\hat{\boldsymbol{z}}$ unto the tangent plane with normal $\hat{\boldsymbol{n}}$ we find that

$$\Delta\boldsymbol{p} = \left(\frac{\Delta z}{1 - n_z^2}\right)\begin{bmatrix} -n_x n_z \\ -n_y n_z \\ 1 - n_z^2 \end{bmatrix}. \tag{31}$$

Clearly, the tangent plane normal, $\hat{\boldsymbol{n}}$, must feature a minimal angle, φ, to $\hat{\boldsymbol{z}}$, such that over the increment Δz the distance traversed by $\Delta\boldsymbol{p}$ is less than or equal to the largest observed grain boundary displacement. Beyond this constraint, without any additional prior information on the grain shape morphology, it is reasonable to assume that the distribution of tangent plane normals, $\hat{\boldsymbol{n}}$, spans the allowable part of the unit ball uniformly. Specifically, we introduce the feasible set of tangent normals, $\hat{\boldsymbol{n}} \in \mathcal{W}_\phi$, defined as

$$\hat{\boldsymbol{n}} \in \mathcal{W}_\varphi \quad \text{if} \quad \hat{\boldsymbol{n}} \in \mathcal{S}^3 \quad \text{and} \quad \arccos(|\hat{\boldsymbol{n}}^T\hat{\boldsymbol{z}}|) > \varphi \tag{32}$$

where $\mathcal{S}^3$ is the shell of the unit ball in $\mathbb{R}^3$. Our postulate then takes the form

$$\hat{\boldsymbol{n}} \sim U(\mathcal{W}_\chi) \tag{33}$$

where U is the uniform distribution.

Given that the tangent plane normal, $\hat{\boldsymbol{n}}$, is a stochastic variable it follows that the vector $\Delta \boldsymbol{p}$ is also a stochastic variable. We can therefore define a x-y planar stochastic displacement vector, $\boldsymbol{u} \in \mathbb{R}^2$, as

$$\boldsymbol{u} = \left(\frac{\Delta z}{1 - n_z^2}\right) \begin{bmatrix} -n_x n_z \\ -n_y n_z \end{bmatrix}. \tag{34}$$

The probability density function (PDF) of $\boldsymbol{u}$ describes a distribution of displacements that originate from the postulated grain boundary curvature. We note that $\boldsymbol{u}$ is isotropic in u_x and u_y with mean $\mathbf{0}$. In fig. S16 B (solid line) the PDF (profile) of $\boldsymbol{u}$ is graphed by drawing 10 000 000 random samples from (34).

Let us now consider the case where an orientation-strain map has been reconstructed together with an accompanying grain boundary map. We defined a measured grain boundary displacement, $\boldsymbol{y} \in \mathbb{R}^2$, by:

1. Selecting a grain boundary point, $\boldsymbol{p}$, in one of the reconstructed z-slices.

2. Computing the candidate displacement vectors between $\boldsymbol{p}$ and all grain boundary points at $z + \Delta z$ (i.e in the consecutive z-slice).

3. Selecting $\boldsymbol{y}$ as the displacement vector with the minimal euclidean norm.

Thus, the measurement, $\boldsymbol{y}$, represents a noisy measurement of the true displacement, $\boldsymbol{u}$. We used an additive error model

$$\boldsymbol{y} = \boldsymbol{u} + \boldsymbol{e}, \tag{35}$$

where $\boldsymbol{e} \in \mathbb{R}^2$ is the sought displacement error variable. When $\boldsymbol{e} \rightarrow 0$ we expect $\boldsymbol{y}$ to be a draw representative of $\boldsymbol{u}$, and when $\boldsymbol{e}$ is large we expect the distribution of $\boldsymbol{y}$ to depart from that of $\boldsymbol{u}$. The grain boundary displacement error, $\boldsymbol{e}$, is here postulated as an aggregated displacement error that stems from, possibly, multiple sources, such as finite data size, data noise, outliers, forward model inconsistency, loss of precision etc. Without any further knowledge we take $\boldsymbol{e}$ to be multivariate Gaussian

$$\boldsymbol{e} \sim \mathcal{N}(\boldsymbol{\mu}, \boldsymbol{\Sigma}) \tag{36}$$

Given a series of measurements $\boldsymbol{y}_1, \boldsymbol{y}_2, ..., \boldsymbol{y}_N$, the task is now to estimate the parameters, $\boldsymbol{\mu}, \boldsymbol{\sigma}$, of the error distribution. Letting $\mathbb{E}[]$ be the mean operator we find that

$$\mathbb{E}[\boldsymbol{e}] = \mathbb{E}[\boldsymbol{y} - \boldsymbol{u}] = \mathbb{E}[\boldsymbol{y}], \tag{37}$$

and Letting $\mathbb{V}[]$ be the variance operator we find that

$$\mathbb{V}[\boldsymbol{e}, \boldsymbol{e}] = \mathbb{V}[\boldsymbol{y}, \boldsymbol{y}] - \mathbb{V}[\boldsymbol{u}, \boldsymbol{u}], \tag{38}$$

where it was used that $\boldsymbol{u}$ is zero mean. To proceed in estimating the error parameters, $\boldsymbol{\mu}, \boldsymbol{\sigma}$, we must first determine the unknown angle φ which defines the distribution of $\boldsymbol{u}$. We approximated φ from displacement data, $\boldsymbol{y}_1, \boldsymbol{y}_2, ..., \boldsymbol{y}_N$, by taking the maximum displacement increment and computing the corresponding tangent plane normal angle needed to achieve this displacement,

$$\varphi = \arctan\left(\frac{|\Delta z|}{\sqrt{\boldsymbol{y}_{max}^T \boldsymbol{y}_{max}}}\right). \tag{39}$$

With $\Delta z = \pm 3\mu$m equation (39) gave φ =7.77^o.

With φ determined the quantities, $\mathbb{V}[\boldsymbol{y}, \boldsymbol{y}]$ and $\mathbb{E}[\boldsymbol{y}]$ involved in equations (37) and (38) were estimated from the displacement data using a maximum likelihood approach. Likewise, we sampled $\boldsymbol{u}$ 10 000 000 times and estimated $\mathbb{V}[\boldsymbol{u}, \boldsymbol{u}]$ numerically using a maximum likelihood approach. The result of this procedure is found in table S1. In fig. S16 the grain boundary skeletons are shown (A) together with the distribution of $\boldsymbol{y}$ and $\boldsymbol{u}$ in (B). We reiterate that, given our model assumptions, in an error free reconstruction, the distributions of $\boldsymbol{y}$ and $\boldsymbol{u}$ should be identical. The spatial errors manifest in fig. S16 B as broadening in distributions of y_x and y_y compared to the postulated error free set of displacements u_x.

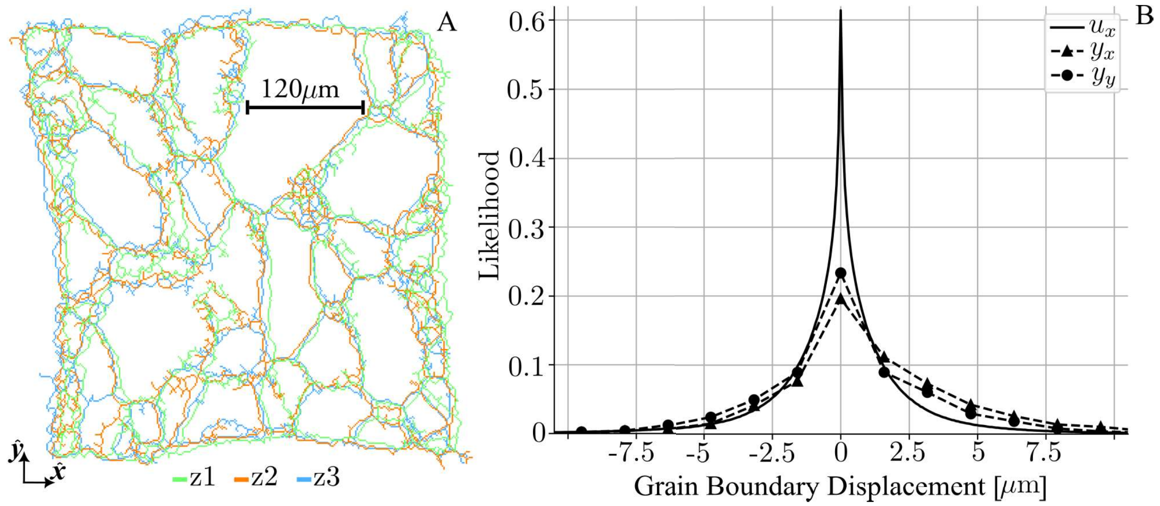

Figure S16: **Grain boundary displacement distributions**. Grain boundary skeletons obtained by morphological thinning of respective KAM-maps for the three reconstructed z-layers have been overlaid in green, orange and blue (A). Distributions over the measured grain boundary displacements, y_x, y_y, are shown in (B) as dashed lines. Due to spatial errors in the reconstruction the distributions of y_x and y_y show broadening compared to the expected set of displacements u_x. This feature was exploited to estimate the spatial error of the reconstruction.

3.2 Stress Resolution

Although we do not have access to a ground truth stress field, it is nevertheless possible to evaluate the accuracy of our reconstructed stress field by comparing to well established theory in the field of continuum mechanics. For a body in static equilibrium, in the absence of body forces, Cauchy's first law of motion demands that the divergence of stress vanish everywhere

$$\text{div}(\sigma_{ij}) = \sum_{j=1}^{j=3} \frac{\partial \sigma_{ij}}{\partial x_j} = 0, \tag{40}$$

where $x_1 = x$, $x_2 = y$ and $x_3 = z$. In our case we have access to a finite approximation of the continuous stress field with one stress tensor per voxel. To evaluate the differential terms in

(40) we must therefore use a finite difference approximation,

$$\begin{aligned}\frac{\partial\sigma_{ij}}{\partial x} &\approx \frac{\sigma_{ij}(x+dx,y,z)-\sigma_{ij}(x,y,z)}{dx},\\ \frac{\partial\sigma_{ij}}{\partial y} &\approx \frac{\sigma_{ij}(x,y+dy,z)-\sigma_{ij}(x,y,z)}{dy},\\ \frac{\partial\sigma_{ij}}{\partial z} &\approx \frac{\sigma_{ij}(x,y,z+dz)-\sigma_{ij}(x,y,z)}{dz}.\end{aligned} \tag{41}$$

To make all voxel dimensions equal we interpolate our reconstructed volume linearly in z such that the finite increment $dx = dy = dz = 1.5\mu$m can be used in (41). Inserting (41) into (40) and multiplying though with dx yields three ($i = 1, 2, 3$) equations

$$\begin{aligned}r_i(\sigma_{ij},x,y,z) = \sigma_{i1}(x+dx,y,z) &- \sigma_{i1}(x,y,z)\\ +\sigma_{i2}(x,y+dx,z) &- \sigma_{i2}(x,y,z)\\ +\sigma_{i3}(x,y,z+dx) &- \sigma_{i3}(x,y,z).\end{aligned} \tag{42}$$

When $r_i(\sigma_{ij},x,y,z) \neq 0$ there exist an error in the stress field reconstruction and we seek to quantify how much the corresponding stress tensor, $\sigma_{ij}(x,y,z)$, need to be perturbed to bring balance of forces. To this end we introduce the local stress increment, $d\sigma_{ij}$, and define a perturbed stress

$$\sigma'_{ij} = \sigma_{ij} + d\sigma_{ij}. \tag{43}$$

From the linearity of (42) it follows that any stress increment that satisfy

$$\begin{aligned}r_i(\sigma'_{ij},x,y,z) = r_i(\sigma_{ij},x,y,z) + r_i(d\sigma_{ij},x,y,z) =\\ r_i(\sigma_{ij},x,y,z) - d\sigma_{i1} - d\sigma_{i2} - d\sigma_{i3} = 0,\end{aligned} \tag{44}$$

will bring balance of forces. To parameterise the solutions, $d\sigma_{ij}$, to equation (44) we the introduce a column vector format

$$d\bar{\boldsymbol{\sigma}} = \begin{bmatrix} d\sigma_{11}\\ d\sigma_{22}\\ d\sigma_{33}\\ d\sigma_{12}\\ d\sigma_{13}\\ d\sigma_{23}\end{bmatrix}, \quad \boldsymbol{r} = \begin{bmatrix} r_1(\sigma_{ij},x,y,z)\\ r_2(\sigma_{ij},x,y,z)\\ r_3(\sigma_{ij},x,y,z)\end{bmatrix}. \tag{45}$$

Equation (44) can now be written as

$$\boldsymbol{r} = \boldsymbol{A}d\bar{\boldsymbol{\sigma}}, \tag{46}$$

where

$$\boldsymbol{A} = \begin{bmatrix} 1 & 0 & 0 & 1 & 1 & 0\\ 0 & 1 & 0 & 1 & 0 & 1\\ 0 & 0 & 1 & 0 & 1 & 1\end{bmatrix}. \tag{47}$$

While (46) has many solutions, only one will uniquely minimise the Euclidean norm of the stress increment, $d\bar{\boldsymbol{\sigma}}$, namely the least squares solution

$$d\bar{\boldsymbol{\sigma}} = (\boldsymbol{A}^T\boldsymbol{A})^{-1}\boldsymbol{A}^T\boldsymbol{r}. \tag{48}$$

By computing $\boldsymbol{r}$ and solving for $d\bar{\boldsymbol{\sigma}}$ at every voxel in the reconstructed stress volume independently, an out of balance stress tensor voxel volume, $\Delta\boldsymbol{\sigma}(x, y, z)$, could be defined. The result of this computation is shown in figs. S17A-F for the central slice (z2). The corresponding histograms over out of balance stress are shown in figs. S17G-L together with histograms of the out of balance stress close to grain boundaries. As shown in Fig. M, we here defined proximity to grain-boundaries by twice dilating the binary grain-boundary skeleton. Voxels close to grain boundaries showed a larger variance compared to the variance of the total distribution. The standard deviation for the respective distributions of Fig. S17 are given in table S2. Comparing to figs. S11-S13, we note that while the absolute standard deviation across stress components are similar (8-12 MPa) the relative error in stress is lower for the axial stress components ($\sigma_{xx}, \sigma_{yy}, \sigma_{zz}$) compared to the shear stress ($\sigma_{xy}, \sigma_{xz}, \sigma_{xz}$).

Close to grain boundaries, multiple distinct grains diffract simultaneously, maximising diffraction peak overlap and making orientation classification challenging. The elevated error in stress close to grain boundaries can therefore be explained, in part, by local orientation reconstruction errors that propagate into stress owing to the anisotropic stiffness model. Beyond this, we emphasises that the true stress fields may feature sharp interfaces close to and at grain boundaries with local stress gradients not fully characterised at a finite spatial resolution.

Table S2: Standard Deviation of Stress Components (MPa)

	$\Delta\sigma_{xx}$	$\Delta\sigma_{yy}$	$\Delta\sigma_{zz}$	$\Delta\sigma_{xy}$	$\Delta\sigma_{xz}$	$\Delta\sigma_{yz}$
Whole Layer	9.76	9.73	7.82	12.28	10.00	10.02
Grain Boundary	13.55	13.71	10.74	16.99	13.99	14.01

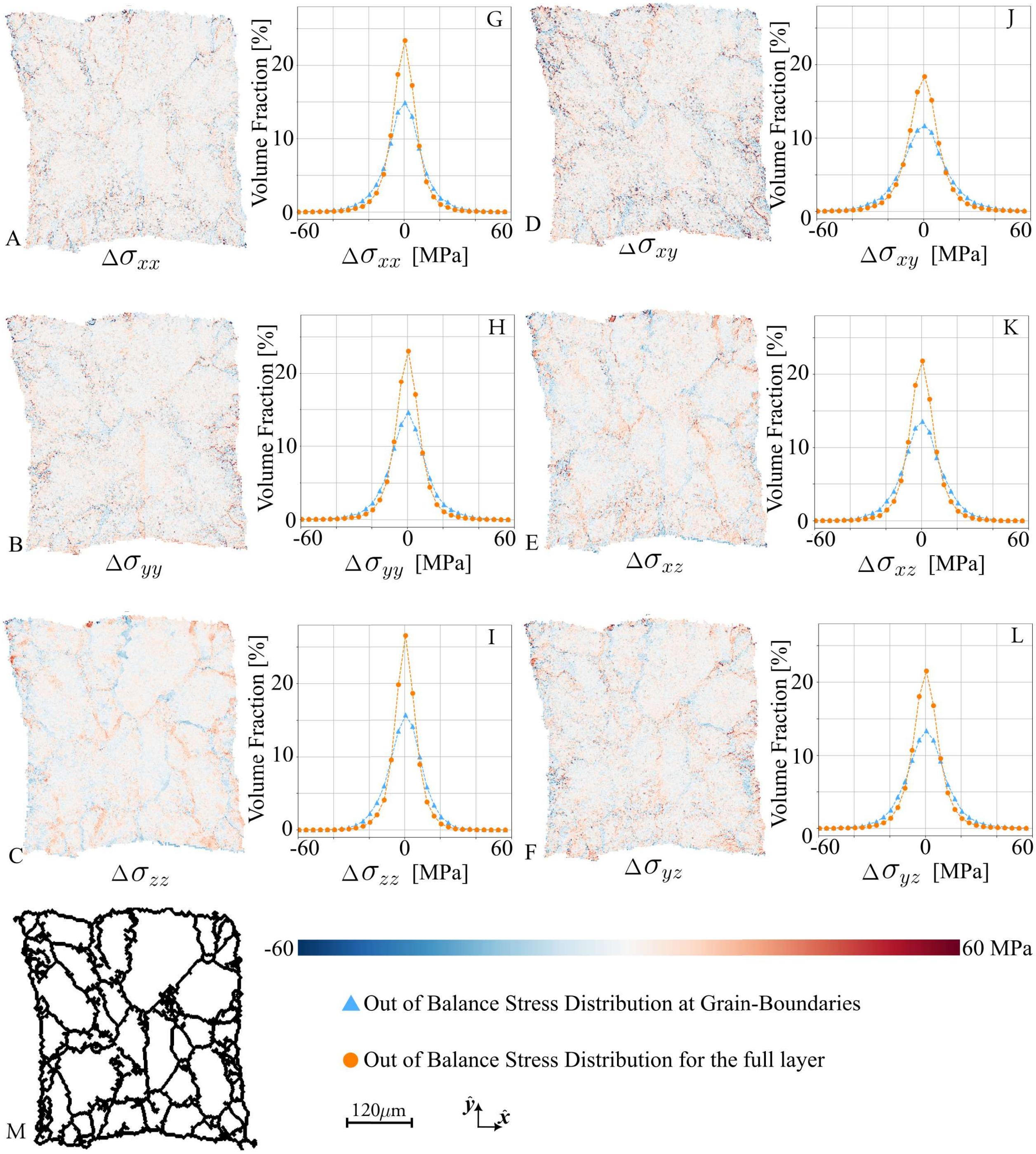

Figure S17: **Residual stress**. Out of balance stress tensor fields (A-F) and corresponding histograms (G-L) for the central slice (z2). Voxels close to grain-boundaries are marked in (M) and show a higher out of balance stress variance in the histograms (G-L). The out of balance stress is a measure of reconstruction error.
